# Bioactive Abietane-Type Diterpenoid Glycosides from Leaves of *Clerodendrum infortunatum* (Lamiaceae)

**DOI:** 10.3390/molecules26144121

**Published:** 2021-07-06

**Authors:** Md. Josim Uddin, Daniela Russo, Md. Anwarul Haque, Serhat Sezai Çiçek, Frank D. Sönnichsen, Luigi Milella, Christian Zidorn

**Affiliations:** 1 Pharmazeutisches Institut, Abteilung Pharmazeutische Biologie, Christian-Albrechts-Universität zu Kiel, Gutenbergstrasse 76, 24118 Kiel, Germany; juddin@pharmazie.uni-kiel.de (M.J.U.); scicek@pharmazie.uni-kiel.de (S.S.Ç.); 2 Department of Pharmacy, International Islamic University Chittagong, Chittagong 4318, Bangladesh; 3 Department of Science, University of Basilicata, Viale dell’ Ateneo Lucano 10, 85100 Potenza, Italy; daiela.russo@unibas.it (D.R.); luigi.milella@unibas.it (L.M.); 4 Spinoff BioActiPlant s.r.l., Viale dell’ Ateneo Lucano 10, 85100 Potenza, Italy; 5 Department of Experimental Pathology, Graduate School of Comprehensive Human Sciences, University of Tsukuba, Ibaraki 305-8575, Japan; a.haque5314@gmail.com; 6 Department of Pharmacy, University of Rajshahi, Rajshahi 6205, Bangladesh; 7 Otto Diels Institute for Organic Chemistry, University of Kiel, Otto-Hahn-Platz 4, 24118 Kiel, Germany; fsoennichsen@oc.uni-kiel.de

**Keywords:** *Clerodendrum infortunatum*, terpenoids, phenylpropanoids, antidiabetic, breast cancer

## Abstract

In this study, two previously undescribed diterpenoids, (5*R*,10*S*,16*R*)-11,16,19-trihydroxy-12-*O*-β-d-glucopyranosyl-(1→2)-β-d-glucopyranosyl-17(15→16),18(4→3)-*diabeo*-3,8,11,13-abietatetraene-7-one (**1**) and (5*R*,10*S*,16*R*)-11,16-dihydroxy-12-*O*-β-d-glucopyranosyl-(1→2)-β-d-glucopyranosyl-17(15→16),18(4→3)-*diabeo*-4-carboxy-3,8,11,13-abietatetraene-7-one (**2**), and one known compound, the C_13_-nor-isoprenoid glycoside byzantionoside B (**3**), were isolated from the leaves of *Clerodendrum infortunatum* L. (Lamiaceae). Structures were established based on spectroscopic and spectrometric data and by comparison with literature data. The three terpenoids, along with five phenylpropanoids: 6′-*O*-caffeoyl-12-glucopyranosyloxyjasmonic acid (**4**), jionoside C (**5**), jionoside D (**6**), brachynoside (**7**), and incanoside C (**8**), previously isolated from the same source, were tested for their in vitro antidiabetic (α-amylase and α-glucosidase), anticancer (Hs578T and MDA-MB-231), and anticholinesterase activities. In an in vitro test against carbohydrate digestion enzymes, compound **6** showed the most potent effect against mammalian α-amylase (IC_50_ 3.4 ± 0.2 μM) compared to the reference standard acarbose (IC_50_ 5.9 ± 0.1 μM). As yeast α-glucosidase inhibitors, compounds **1**, **2**, **5**, and **6** displayed moderate inhibitory activities, ranging from 24.6 to 96.0 μM, compared to acarbose (IC_50_ 665 ± 42 μM). All of the tested compounds demonstrated negligible anticholinesterase effects. In an anticancer test, compounds **3** and **5** exhibited moderate antiproliferative properties with IC_50_ of 94.7 ± 1.3 and 85.3 ± 2.4 μM, respectively, against Hs578T cell, while the rest of the compounds did not show significant activity (IC_50_ > 100 μM).

## 1. Introduction

*Clerodendrum* (Lamiaceae) is a diverse genus with about 580 species [1] of small trees, shrubs, or herbs, mostly distributed throughout tropical and subtropical regions of the world [2]. *Clerodendrum infortunatum* L. (Syn.: *Clerodendrum viscosum* Vent), locally known as Bhat, is a terrestrial shrub with a noxious odor, distributed throughout mixed deciduous and evergreen to semi-evergreen forests of Bangladesh and the Indian state of West Bengal [3]. Due to its easy availability and presumed beneficial activities, various parts of the plant, particularly the leaves and roots, are extensively used in Indian and Bangladeshi traditional medicine for some common ailments. In folk medicine, the leaves and roots are used to cure helminthiasis, tumors, skin diseases, snakebites, and scorpion stings. Infusions of the leaves are also used as a bitter tonic and antiperiodic for the treatment of malaria. The freshly extracted juice of the leaves is considered to be a good laxative and cholagogue [4,5]. Some experimental evidence has proven the traditional claims, showing various biological effects, such as anti-snake venom activity [6], analgesic and anticonvulsant activities [7], nootropic activity [8], antimicrobial activity [9], antioxidative potential [10], and hepatoprotective activity [11]. Earlier, phytochemical studies of *C. infortunatum* leaves revealed the presence of flavonoids, phenolic compounds, terpenoids, steroids, and phenylpropanoids [12].

Due to the increasing life expectancy worldwide, the prevalence of age-associated diseases (including cardiovascular disease, cancer, type 2 diabetes, neurodegenerative disorders, osteoporosis, pancreatitis, and hypertension) is rising exponentially with age. Consequently, the treatment and prevention of these conditions is turning into a priority in medicine, due to the rapid increase of elderly populations, particularly in Western countries [13,14]. Natural products remain a rich source of anticancer, antidiabetic, and anti-neurodegenerative disorder agents; more than half of all drugs used for the treatment of cancer are either natural products or derived from natural products [15].

Terpenoids, abundant in medicinal plants, structurally constitute complex and diverse groups of natural products. Naturally occurring terpenoids have been shown to have significant preventive properties against age-related diseases such as tumors, diabetes, inflammation, cardiovascular diseases, and neurodegenerative disorders [16].

Phenylpropanoids are a group of natural products with widespread distribution in plants, and are also considered to be potential agents against age-related disorders due to possessing anticancer, antidiabetic, neuroprotective, cardioprotective, antimicrobial, antioxidative, and enzyme inhibitory activities at comparatively low concentrations [17,18].

In view of the important role of terpenoids and phenylpropanoids in treating age-related disorders, eight bioactive compounds, including five previously isolated phenylpropanoids, were investigated for their antiproliferative and antimetastatic effects by two human triple negative breast cancer (TNBC) cell lines (Hs578T and MDA-MB-231). Additionally, their anti-diabetic properties were assessed through α-amylase and α-glucosidase enzyme inhibition, and their cholinesterase inhibitory properties were also assessed.

## 2. Results and Discussion

### 2.1. Phytochemical Investigation

**Figure 1:**
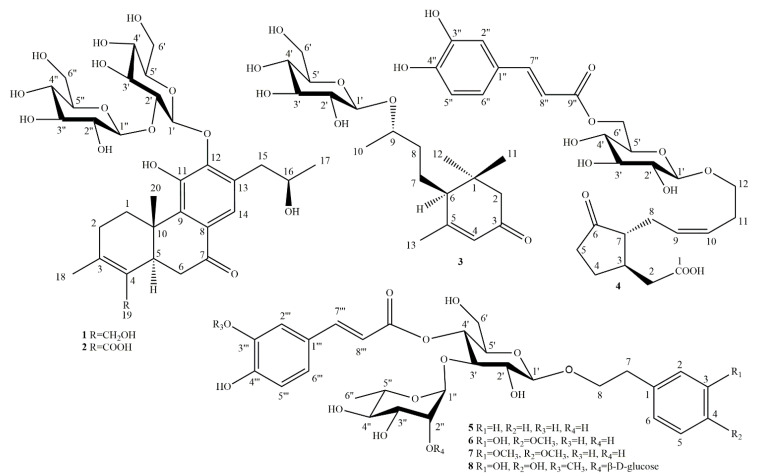
Structures of compounds **1**–**8** isolated from leaves of *Clerodendrum infortunatum*.

In the present study, we analyzed the specialized natural products from *C. infortunatum* leaves, resulting in the isolation and structural characterization of three terpenoids, including two previously undescribed diterpenoids. A butanol fraction of acetone extract of *C. infortunatum* leaves was subjected to open column chromatography using a silica gel and subsequent semi-preparative HPLC with reversed phase column, and the abietanes (**1** and **2**) were obtained as amorphous solids, together with the previously reported C_13_ nor-isoprenoid glycoside (**3**) (Figure 1). In our previous study, five phenylpropanoid glycosides: 6′-*O*-caffeoyl-12-glucopyranosyloxyjasmonic acid (**4**), jionoside C (**5**), jionoside D (**6**), brachynoside (**7**)**,** and incanoside C (**8**) were reported from the same source (Figure 1) [12].

Structures (**1**–**3**) were identified based on the ^1^H, ^13^C NMR, and high-resolution mass spectrometry data (Figures S1–S13). Based on their spectra, the isolates were found to be novel abietane glycosides (**1** and **2**) with a sophorose moiety at C-12.

Compound **1** was obtained as a brown powder, and its molecular formula C_32_H_46_O_15_ was confirmed by HR-ESI-MS (*m*/*z* = 669.2763 [M − H]^−^). In the ^1^H NMR spectrum (Table 1), compound **1** showed the presence of one aromatic proton at δ_H_ 7.42 (1H, s), which was assumed to be located in the para position, and suggested one penta-substituted benzene ring. The ^1^H NMR also exhibited two anomeric protons at δ_H_ 4.64 (1H, *d*, *J* = 8.0 Hz), and 4.75 (1H, *d*, *J* = 8.0 Hz); a methine at δ_H_ 2.83 (1H, m); an oxygenated methine at δ_H_ 3.97 (1H, *dd*, *J* = 12.5, 6.0 Hz); two oxygenated methylene protons at δ_H_ 3.85 (1H, *d*, *J* = 12.0 Hz) and δ_H_ 4.13 (1H, *d*, *J* = 12.0 Hz); four methylene groups at δ_H_ 1.43 (1H, *td*, *J* = 12.5, 6.5 Hz) and 3.31 (1H, *dd*, *J* = 13.0, 6.5 Hz), 2.00 (1H, *dd*, *J* = 18.0, 6.0 Hz) and 2.20 (1H, *t*, *J* = 10.0 Hz), 2.66 (1H, *dd*, *J* = 13.5, 6.0 Hz) and 2.92 (1H, m), 2.90 (1H, m) and 2.96 (1H, *t*, *J* = 3.0 Hz); three methyl groups at 1.70 (3H, *d*, *J* = 2.0 Hz), 1.18 (3H, s), and 1.00 (3H, *d*, *J* = 6.0 Hz).

The ^13^C NMR spectrum (Table 2) revealed the presence of a quaternary carbon, indicated by a signal at δ_C_ 197.8, typical of a ketone; one 8,9,11,12,13-pentasubstituted benzene ring supported by signals at δ_C_ 128.3, 137.2, 147.8, 148.4, 131.4, respectively; two anomeric carbons displaying the same shifts at δ_C_ 103.6; two methine carbons at δ_C_ 42.7 and 65.5; five methylenes at δ_C_ 31.1, 29.7, 57.5, 36.6, and 39.2. An olefinic moiety was deduced from signals at δ_C_ 129.9 (C-3) and 129.2 (C-4).

Further, ten oxygenated aliphatic carbons (δ_C_ 80.8, 76.1, 69.5, 69.9, 61.1, 74.1, 76.2, 77.5, 77.4, and 60.9) together with two anomeric carbons at δ_C_ 103.6 reflected the presence of two glucose units. The coupling values of both anomeric protons (*J*_1′–2′_ = 8.0 Hz, *J*_1″–2″_ = 8.0 Hz) indicated that the sugar chains of **1** were glucopyranosyl-(1→2)-glucopyranosyl, and that both anomeric protons were in β-position. This was confirmed by HMBC data, and thus the linkage of the β-d-sophoroside in position C-12 was also established.

**Figure 2:**
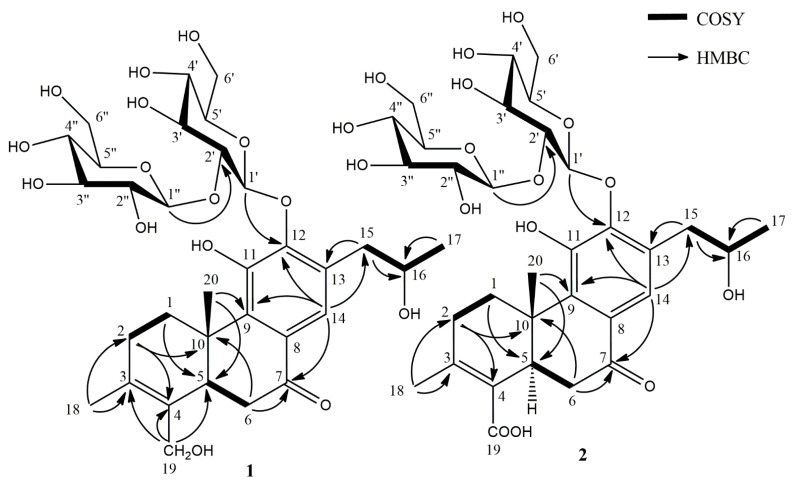
Main ^1^H-^1^H COSY and HMBC correlations of compounds **1** and **2**.

In the HMBC spectrum, correlations between H-1′ (δ_H_ 4.64) and C-12 (δ_C_ 148.4), and H-15 (δ_H_ 2.66) and C-13 (δ_C_ 131.4) were observed (Figure 2), which proved that the glucose unit and the propanol moiety were connected to the benzene ring via C-12 and C-13, respectively.

A majority of the known plant-derived abietane-type diterpenes possess the same carbon skeleton, displaying a trans-fused system of two six-membered rings, a β-oriented methyl at C-10, and α-orientation of the proton at C-5 [19]. In order to identify the absolute configuration of C-16, the chemical shifts at C-15, C-16, and C-17 were compared with that of three known compounds, szemaoenoid A, szemaoenoid C [20], and (5*R*,10*S*,16*R*)-11,16-dihydroxy-12-methoxy-17(15→16)-abeoabieta-8,11,13-trien-3,7-dione [21]. Structures of these three compounds had been established by Mosher esterification and X-ray crystallography (Table 3). In view of the identical NMR data, the absolute configuration of C-16 of **1** was assigned as *R* conformation. Therefore, based on these findings and the supporting correlations along with the identical NMR data and biogenesis, compound **1** was established as (5*R*,10*S*,16*R*)-11,16,19-trihydroxy-12-*O*-β-d-glucopyranosyl-(1→2)-β-d-glucopyranosyl-17(15→16),18(4→3)-*diabeo*-3,8,11,13-abietatetraene-7-one, a previously undescribed natural product.

Compound **2** was isolated as a colorless amorphous powder. The molecular formula of **2** was deduced as C_32_H_44_O_16_ based on the HR-ESI-MS (*m*/*z* = 683.2556 [M − H]^−^), which has one COOH instead of CH_2_OH at the same position as that of **1.** An abietane-type diterpenoid derivative was evident based on its UV maxima at 219, 273, 319 nm, and NMR data. Analysis of the ^1^H and ^13^C NMR data of **2** (Table 1 and Table 2) revealed similar substituent patterns to that of **1**, except a carboxylic group at C-19 rather than the methyleneoxy group. The ^1^H and ^13^C NMR data assignments were based on ^1^H-^1^H, COSY, HSQC, and HMBC spectra (Figures S7–S11). The ^13^C NMR spectrum displayed signals for a ketone group at δ_C_ 202.9, a carboxylic group at δ_C_ 170.7, two tertiary methyl groups, and four double bonds including an aromatic ring characteristic of an abieta- 3,8,11,13-tetraene.

Comparing NMR data of **2** with **1** revealed that the aglycon part of both compounds was linked with the same sugar moiety. Based on 1D and 2D spectra, all sugar protons and carbons were assigned β-d-sophorose. Besides NMR spectra, acidic hydrolysis employing GLC-MS/MS analysis of both compounds **1** and **2** also confirms two β-d-glucose units as its sugar component (Section 3.9). From the biogenetic considerations and identical NMR data, **2** was inferred as possessing an identical absolute configuration to **1**. Thus, compound **2** was identified as (5*R*,10*S*,16*R*)-11,16-dihydroxy-12-*O*-β-d-glucopyranosyl-(1→2)-β-d-glucopyranosyl-17(15→16),18(4→3)-*diabeo*-4-carboxy-3,8,11,13-abietatetraene-7-one, a previously undescribed natural product.

The structure of the known C_13_-nor-isoprenoid glycoside was confirmed as byzantionoside B (**3**) by spectrometric and spectroscopic methods (HR-ESI-MS, and ^1^H NMR, ^13^C NMR, COSY, HSQC, HMBC), and by comparison with literature data [22,23].

### 2.2. α-Amylase and α-Glucosidase Inhibition

α-Amylase (pancreatic enzyme) and α-glucosidase (intestinal enzyme) inhibitors reduce the conversion of carbohydrates into monosaccharides and are considered adjunctive therapeutics for the treatment of diabetes mellitus type 2. Natural products displaying α-amylase and α-glucosidase inhibitory properties could therefore be beneficial for the management of diabetes and obesity by controlling peak blood glucose levels. Compounds **1**–**3** (terpenoids) and **4**–**8** (phenylpropanoids) (Figure 1) demonstrated α-amylase and α-glucosidase inhibition, reflecting their previous records (Table 4) [16,18]. One terpenoid and one phenylpropanoid were found to have a significant mammalian α-amylase and yeast α-glucosidase inhibition compared to acarbose, a drug to treat type 2 diabetes mellitus, which was used as a positive control. In the α-amylase inhibition assay, compound **6** showed the most potent activity (IC_50_ 3.4 ± 0.2 μM), and it was found to be almost two times more active than acarbose (IC_50_ 5.9 ± 0.1 μM). Compounds **4**, **1**, **8**, and **5** displayed a slightly lower potency, ranging from IC_50_ 13.0–24.9 μM, which was comparable to that of acarbose, while compounds **3** and **7** were almost inactive.

Acarbose, a clinically used glycosidase inhibitor, usually demonstrates a weak inhibitory effect against yeast α-glucosidase compared to mammalian glycosidases. Therefore, by using acarbose as a positive control in the yeast α-glucosidase assay (IC_50_ 665 ± 42 μM), compounds **1**, **2**, **5**, and **6** were established as moderate α-glucosidase inhibitors, with IC_50_ values ranging from 24.6 to 96.0 μM (Table 4). The remaining four compounds showed no activity against yeast α-glucosidase at the tested concentrations.

Phenylpropanoid glycosides and abietane diterpenoids have previously been reported as being active against α-glucosidase and α-amylase [24,25]. The number and positions of hydroxy groups on natural compounds are crucial structural features to understand their enzyme inhibition [26]. In our compounds, the presence of one additional hydroxy group at C-4 at compound **6** seems to be involved in the more pronounced α-amylase inhibitory activity in comparison with compound **7**, where the methoxy substituents and sugar moiety could affect negatively on the inhibitory activity [27,28].

### 2.3. Cholinesterase Inhibitory Properties

Alzheimer’s disease (AD) displays low levels of acetylcholine due to neurons degeneration, for this reason accpted therapeutic strategies for a symptomatic treatment of this illness include cholinesterases, acetylcholinesterase (AChE) and butyrylcholinesterase (BChE) inhibitors, as galanthamine. These enzymes are responsible for acetylcholine’s hydrolysis, which plays an essential role in the proper functioning of the central cholinergic system, respectively. Due to having antioxidant, antiaging, and neuroprotective properties, terpenoids and phenylpropanoids were tested for their effects in managing AD [16,18]. However, from the tested compounds, **1**, **2**, **5**, and **8** displayed only low AChE inhibitory effects ranging from IC_50_ values of 139–191 μM, while **3, 4, 6,** and **7** showed no inhibitory activity on AChE in the tested concentration range. None of the tested compounds demonstrated any activity towards BChE. Galanthamine was used as a positive control for both the AChE (IC_50_ 2.9 ± 0.5 μM) and BChE (IC_50_ 23 ± 2 μM) inhibition assays (Table 4).

The presence of sugar moiety in compounds may interfere with their ChE inhibitory activities, which modify the affinities toward enzymes [28]. Comparing our results with the activity of abietane diterpenoids isolated from *Caryopteris mongolica*, it implies that the presence of sugar moiety in tested compounds might reduce their inhibitory activity [21].

### 2.4. Antiproliferative and Cytotoxic Activities

Triple-negative breast cancers are a highly aggressive, heterogeneous subtype of breast cancer, with a poor survival rate. These breast cancers are characterized by a lack of expression of estrogen and progesterone receptors as well as a lack of amplification human epidermal growth factor receptor 2 [29]. There are no approved targeted therapies for triple-negative breast cancers, because they do not respond to available targeted therapies. However, patients usually receive chemotherapy with cytotoxic agents such as taxanes [30].

Terpenoids and phenylpropanoids are well-known for their cytotoxic and anticancer activity [31,32,33]. Compounds **1**–**8** were therefore tested for their cytotoxicity and effects on the cell migration of Hs578T and MDA-MB-231, which are triple negative breast cancer cell lines. Among the tested compounds, the concentration-related cytotoxic responses were observed with IC_50_ values of 85.3 ± 2.4 and 96.5 ± 1.5 μM against Hs578T and MDA-MB-231, respectively for compound **5**, and 94.7 ± 1.3 μM against Hs578T for compound **3** (Table 5). The rest of the compounds did not show significant activity within the tested range (IC_50_ > 100 μM).

#### 2.4.1. Effects on TNBC Cell Proliferation

**Figure 3:**
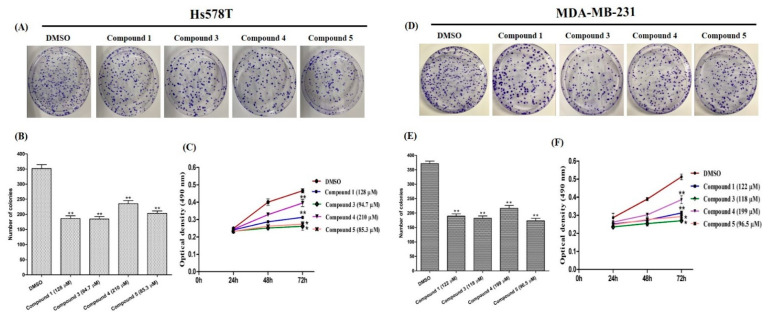
Inhibition of TNBC cell proliferation. (**A**,**D**): visualized colonies after two weeks of treatment of TNBC cells with tested compounds. (**B**,**E**): quantified colonies from colony formation assay. (**C**,**F**): the amount of viable cells after treatment of the cells with compounds for 24, 48, and 72 h, determined using an MTS assay. Data are presented as the mean ± SD of three independent experiments. Bars and lines with asterisks indicate significant differences from the control at *p* ≤ 0.05 (*) or *p* ≤ 0.01 (**).

To explore the antiproliferative activity of compounds tested in the TNBC cell lines Hs578T and MDA-MB-231, colony formation assays were employed. To fix the effective concentration, the half maximal inhibitory concentration (IC_50_) of each compound was determined (Table 5), and was used as a working concentration for all experiments. In the cell proliferation assay, the compounds triggered a significant reduction in the number of colony formations compared to that of the control (DMSO-treated) cells (Figure 3A,D). The quantified colonies are represented in bar graphs at Figure 3B,E). Moreover, the cell viability outcome also justified the antiproliferative activity of the tested compounds in the MTS assay (Figure 3C,F). The obtained data revealed that compounds **3** and **5** possess moderate antiproliferative effects: they reduced the number of TNBC cell Hs578T (44 and 42%, respectively) and MDA-MB-231 (48 and 43%, respectively) after three days of treatment at IC_50_. Chemotherapeutic compounds interrupt the signaling pathways of cancer and control accelerated proliferation to induce cancer cell death [34]. Natural products are considered a key source in the search for new anticancer compounds [15]. This study displayed that tested compounds moderately suppressed breast cancer cell proliferation and viability.

#### 2.4.2. Effects on Cell Migration

**Figure 4:**
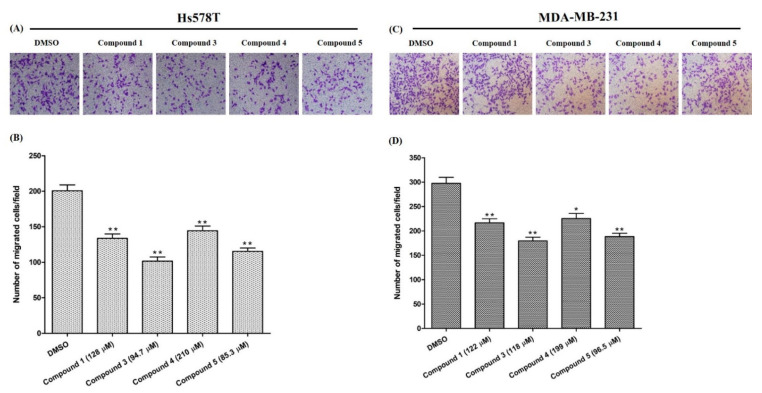
Attenuation of TNBC cell migration. (**A**,**C**): visualized migrated cells after treating with compounds. (**B**,**D**): the migrated cells stained with crystal violet, and photographs taken for the inhibition calculation. The results are presented as the mean ± SD of three independent experiments. Bars with asterisks indicate significant differences from the control at *p* ≤ 0.05 (*) or *p* ≤ 0.01 (**).

The migration of cancer cells are critical determinant steps of tumor metastasis. To evaluate the anti-metastatic effect on breast cancer cells, the inhibition of the cell migration rate is a reliable indicator. Figure 4 shows the inhibition ability of the tested compounds on the migration of the breast cancer cells compared to control cells in DMSO (*p* < 0.01). All tested compounds inhibited the migration of Hs578T cell slightly more than MDA-MB-231. Compound **3** and **5** displayed an interesting activity profile: they were able to inhibit 50% and 43% of the migration of Hs578T cell, and 40% and 37% for MDA-MB-231, respectively, at IC_50_. Cancer metastasis, a multistep process, is a major cause of cancer-associated mortalities. During this process, cancer cells escape and travel from the primary tumor site to a distant area through various cascades of events such as cell adhesion, cell motility and invasion, cell movement, and degradation of the cellular matrix [35,36]. The inhibition of cancer cell migration is a novel strategy for the treatment of metastatic cancers. Our results showed that compounds **3** and **5** effectively suppressed breast cancer cell migration.

#### 2.4.3. Effects on Tumor-Sphere Formation

**Figure 5:**
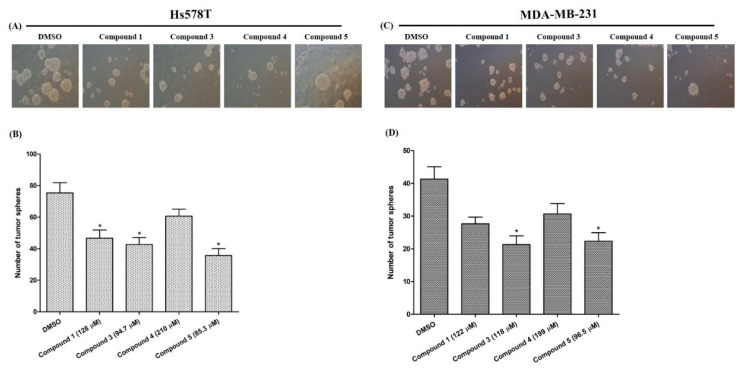
Reduction of the formation of the tumor-sphere. (**A**,**C**): the captured tumor-spheres after five days of treatment with tested compounds. (**B**,**D**): the number of tumor-spheres. Data are presented as the mean ± SD of three independent experiments. The bars with asterisks indicate significant differences from the control at *p* ≤ 0.05 (*).

The capability of compounds to reduce cell size is considered a good indicator in cancer therapy. The in vitro tumor-sphere formation tests demonstrated that compound **5** reduced the cell size (Figure 5A,C) and the cell number significantly (Figure 5B,D) in both cell lines. In vitro tumor-sphere formation is a frequently used new and inexpensive method considered a potential alternative for in vivo screening of anticancer drugs [37]. In the present study, the number and size of tumor spheres were sharply reduced by the compound **5**. However, more intensive research is needed to find out the mechanism of these activities in relation with the respective compound.

## 3. Materials and Methods

### 3.1. General Experimental Procedures

The NMR spectra, 1D (^1^H, ^13^C) and 2D (COSY, HMQC, HMBC), were acquired at 600 MHz on a Fourier transform-NMR “Avance III 600” spectrometer equipped with a cryogenically cooled triple resonance Z-gradient probe head operating at 300 K and pH 7.5 (Bruker BioSpin GmbH, Rheinstetten, Germany). TMS was used as the internal reference standard where chemical shifts reported as δ values. ESI-MS data were obtained via Nexera X2 system (Shimadzu, Kyoto, Japan) connected to an autosampler, column heater, PDA, and a Shimadzu LC-MS 8030 Triple Quadrupole Mass Spectrometer. HR-ESI-MS spectra were recorded on a Q-Exactive Plus spectrometer (Thermo Scientific, Bremen, Germany).

UHPLC experiments were performed employing a VWR Hitachi Chromaster UltraRS liquid chromatograph equipped with ELSD 100 and DAD 6430 detectors. A column, Phenomenex Luna Omega C_18_, 1.6 μm, 100 × 2.1 mm, was used for all the analyses, with the following settings: mobile phase A: 0.1% formic acid in water; mobile phase B: 100% acetonitrile (linear gradient: 0 min 5% B, 35 min 40% B, 50 min 95% B, 60 min 95% B, 60.1–70 min 5%); flow rate: 0.20 mL/min; injection volume: 2 μL; oven temperature: 30 °C (Figure S14). 

Semi-preparative HPLC was performed on a Waters HPLC system (Waters) equipped with Waters Alliance e2695 Separations Module, Alliance 2998 detector, WFC III fraction collector (Waters, Milford, MA, USA), and VP Nucleodur C18 column (250 × 10 mm, 5 μm particle size, Macherey-Nagel, Düren, Germany). Column chromatography was performed with silica gel (40–63 μm; 230–400 mesh, Carl Roth GmbH, Karlsruhe, Germany) and Sephadex LH-20 (GE Healthcare AB, Uppsala, Sweden). Thin-layer chromatography (TLC) was carried out on precoated TLC plates (Silica gel 60 F_254_, Merck, Darmstadt, Germany) using ethyl acetate-water-acetic acid-formic acid (15:5:2:2) as the mobile phase, and the spots were visualized by heating after vanillin-sulphuric acid spray.

### 3.2. Chemicals

Acetylcholinesterase from electric eel (*Electrophorus electricus*, type VI-s, lyophilized powder), acetylthiocholine iodide (ATCI), butyrylcholinesterase from equine serum (lyophilized powder), butyrylthiocholine iodide (BTCI), 5,5′-dithio-bis2-nitrobenzoic acid (DTNB), α-amylase from porcine pancreas, α-glucosidase from *Saccharomyces cerevisiae*, 4-*p*-nitrophenyl-α-D-glucopyranoside, starch, galanthamine, and acarbose were purchased from Sigma-Aldrich (St. Louis, MO, USA). Trizma hydrochloride (Tris-HCl) and bovine serum albumin (BSA) were obtained from Sigma–Aldrich (Steinheim, Germany). Deionized water was produced using a Milli-Q water purification system (Millipore, Bedford, MA, USA). Dulbecco’s Modified Eagle Medium (DMEM) was collected from Gibco (Japan), and fetal bovine serum (FBS) was also collected from Gibco (Waltham, MA, USA). Insulin and penicillin G/streptomycin were collected from Wako (Fujifilm Wako Pure Chemical Corporation, Osaka, Japan), the MTS kit was collected from Promega (Madison, WI, USA), and poly-2-hydroxyethyl methacrylate was collected from Sigma-Aldrich (Taufkirchen, Germany).

### 3.3. Plant Material

The leaves of *C. infortunatum* were collected from Pabna, Bangladesh, in March 2018 at 23 m above mean sea level (coordinates: N 24°03′45.0″; E 89°04′14.0″). Prof. Dr. A.H.M. Mahbubur Rahman, Department of Botany, University of Rajshahi, Bangladesh carried out the botanical identification [38]. A voucher specimen (CV-20180321-04) was deposited in the Department of Botany, University of Rajshahi, Bangladesh.

### 3.4. Extraction and Isolation

The air-dried fresh leaves of *C. infortunatum* (1.15 kg) were powdered and subjected to cold extraction with acetone (5 L) at room temperature five times, for one day each time. The obtained solution was combined, filtered, and evaporated under reduced pressure at 35 °C, yielding 58.0 g of crude acetone extract. The concentrated extract was solvated in a solution of water:methanol (2:1) and partitioned with ethyl acetate and *n*-butanol, respectively, resulting in the ethyl acetate (35.7 g), *n*-butanol (15.0 g), and water (7.30 g) fractions. The butanol extract (15.0 g) was chromatographed to a silica gel column chromatography (CC) eluted with a gradient of increasing methanol (0–100%) in dichloromethane to attain 14 fractions (CV 1 to CV 14).

Fraction CV 14 was subjected to chromatographic separation by Sephadex LH-20 gel column (3 × 100 cm) eluted with methanol to yield eight subfractions (CV 14 A-H). Subfraction CV 14C was treated by semi-preparative HPLC on an RP-18 column (VP 250 × 10 mm Nucleodur C18, 5 μm, flow rate: 2 mL/min) using 0.025% formic acid in water, developing methanol:water solvent mixtures (40:60, isocratic) that yielded three pure compounds: 2 (6.0 mg, *t*_R_ 18 min), 1 (8.5 mg, *t*_R_ 30 min), and 3 (3.0 mg, *t*_R_ 39 min).

(4a*S*,10a*R*)-6-(((2*S*,3*S*,4*S*,6*S*)-4,5-dihydroxy-6-(hydroxymethyl)-3-(((2*S*,3*S*,4*S*,5*S*,6*R*)-3,4,5-trihydroxy-6-(hydroxymethyl)tetrahydro-2*H*-pyran-2-yl)oxy)tetrahydro-2*H*-pyran-2-yl)oxy)-5-hydroxy-1-(hydroxymethyl)-7-((*R*)-2-hydroxypropyl)-2,4a-dimethyl-4,4a,10,10a-tetrahydrophenanthren-9(3*H*)-one (**1**). Brown powder; [α]^25^_D_ +0.035 (MeOH); For ^1^H NMR (methanol-*d4*, DMSO-*d_6_*, 600 MHz) and ^13^C NMR (methanol-*d4*, DMSO-*d_6_*, 150 MHz) data, see Table 1 and Table 2. HR-ESI-MS *m*/*z* 669.2763 [M − H]^−^ (calcd. for C_32_H_46_O_15_, 669.2758). 

(4a*S*,10a*R*)-6-(((3*S*,4*S*,6*S*)-4,5-dihydroxy-6-(hydroxymethyl)-3-(((2*S*,3*S*,4*S*,5*S*,6*R*)-3,4,5-trihydroxy-6-(hydroxymethyl)tetrahydro-2*H*-pyran-2-yl)oxy)tetrahydro-2*H*-pyran-2-yl)oxy)-5-hydroxy-7-(2-hydroxypropyl)-2,4a-dimethyl-9-oxo-3,4,4a,9,10,10a hexahydrophenanthrene-1-carboxylic acid (**2**). Pale brown powder; [α]^25^_D_ +0.030 (MeOH); For ^1^H NMR (methanol-*d4*, DMSO-*d_6_*, D_2_O, 600 MHz) and ^13^C NMR (methanol-*d4*, DMSO-*d_6_*, D_2_O, 150 MHz) data, see Table 1 and Table 2. HR-ESI-MS *m*/*z* 683.2556 [M − H]^−^ (calcd. for C_32_H_46_O_15_, 683.2551).

### 3.5. α-Amylase Inhibition Assay

*α*-Amylase inhibitory activity was determined following the starch-iodine method [39] with some modifications. A 1% starch solution was prepared by 1 g of starch in 10 mL of distilled water following gentle boiling and cooling into 100 mL. A reaction mixture, 25 μL sample (0–1 mM) and 50 μL α-amylase (5 U/mL) in phosphate buffer, was incubated at 37 °C for 10 min. Afterwards, the starch (100 μL, 1% *w*/*v*) solution was added to the mixture and incubated again at 37 °C for 10 min. The enzymatic reaction was suspended by adding HCl (25 μL, 1 N) followed by the incorporation of 50 μL of iodine reagent (2.5 mM I_2_ and 2.5 mM KI). After adding the iodine/iodide solution, based on the colour change, the absorbance was monitored at 630 nm for 10 min. Acarbose was used a positive control. The percentage of inhibition was calculated and results were expressed as IC_50_ (µM).

### 3.6. α-Glucosidase Inhibition Assay

To assess the inhibitory activity of the tested compounds on *α*-glucosidase, all solutions were prepared according to the previously described method [39]. Different concentrations (0–1 mM) of the sample (50 μL) and *α*-glucosidase enzyme (40 μL, 0.1 U/mL) dissolved in phosphate buffer were incubated at 37 °C for 10 min. After combining the substrate 4-*p*-nitrophenyl-α-d-glucopyranoside (40 μL, 2.5 mM) to the enzyme mixture, it was incubated again at 37 °C for 10 min. Na_2_CO_3_ (100 μL, 0.2 M) was used to stop the enzymatic reaction. The release of glucose and *p*-nitrophenol (yellow) was detected spectrophotometrically at 405 nm. Acarbose was used as positive control and the results were expressed as IC_50_ (µM).

### 3.7. Determination of Cholinesterase Inhibitory Activities

The cholinesterase inhibitory (AChE/BChE) properties were ascertained based on Ellman’s method, as previously described [40]. The enzyme activity was detected by spectrophotometric exposure (405 nm), with increasing yellow colour produced from thiocholine, while it reacted with 5,5′-dithio bis-2 nitrobenzoate ions (DTNB). In the AChE inhibitory assay, 25 µL of sample solution (0–1 mM) along with 50 µL of buffer B (50 mM Tris-HCl, pH 8 containing 0.1% BSA), 125 µL of DTNB (3 mM), and 25 µL of 0.05 U/mL AChE were incubated at 37 °C for 10 min. After incubation, 25 µL of acetylthiocholine iodide (5 mM) as AChE substrate was incorporated to the solution. The BChE activity was determined following the same protocol using 25 µL of 5 mM *S*-butyrylthiocholine chloride as BChE substrate and 0.05 U/mL BChE as enzyme. The inhibitory abilities of the compounds (1–8) were assessed at different concentrations. Galanthamine (dissolved in 10% DMSO in methanol) was used as a positive control, while 10% DMSO in methanol was used as a negative control for both assays. The percentage of inhibition was calculated and the results were expressed as IC_50_ (µM).

### 3.8. Determination of Anticancer Activities

#### 3.8.1. Cell Lines and Culture Condition

The human TNBC cell lines Hs578T and MDA-MB-231 were obtained from the American Type Culture Collection and cultured in Dulbecco’s Modified Eagle Medium (DMEM) supplemented with fetal bovine serum (FBS) (10% *v*/*v*), insulin (Hs578T cell only), and penicillin G/streptomycin 1% (*v*/*v*) at 37 °C under 5% CO_2_. The absence of culture contamination by *Mycoplasma* species was confirmed before the experiments.

#### 3.8.2. Colony Formation Assay

The breast cancer cell proliferation activity of the tested compounds was assessed using a colony formation assay [41]. Approximately 400 viable Hs578T and MDA-MB-231 cells were seeded in a 10 cm culture plate containing DMEM medium without or with the tested compounds (at their respective IC_50_ concentration), and incubated for 2 weeks. After incubation, the medium was discarded, and the colonies were washed twice with phosphate-buffered saline (PBS). Subsequently, the colonies were fixed with 4% paraformaldehyde and stained with crystal violet solution. Colonies consisting of more than 20 individual cells were counted by ImageJ software.

#### 3.8.3. Cell Viability Assay

The activity of the selected compounds on breast cancer cell viability were determined employing the MTS assay [42]. Briefly, 5 × 10^3^ cells were seeded in each well of a 96-well plate for 24 h, and growth medium containing different compounds was added and incubated in different periods. A total of 20 µL of the MTS kit was added to each well, and the cells were incubated for 2 h. After incubation, the absorbance was measured at 490 nm with an enzyme-linked immunosorbent assay microplate reader (BioTek Instruments, USA). The absorbance value is directly proportional to the number of living cells.

#### 3.8.4. Transwell Cell Migration Assay

The effects of the selected compounds on breast cancer cell migration were evaluated in transwell chambers according to a published protocol [42]. Briefly, the Hs578T and MDA-MB-231 cells were treated with the tested compounds for 24 h, trypsinized, and washed twice with serum-free medium. Approximately 3 × 10^4^ pretreated cells, suspended in 100 μL of the serum-free medium, were seeded to the upper chamber. The lower chambers were filled with approximately 500 μL of DMEM medium with 10% FBS and incubated for 12 h. After incubation, the cells from the upper surface were wiped off with cotton swabs, while the migrated cells on the opposite side of the transwell were washed twice with PBS, fixed with 4% paraformaldehyde, and finally stained with crystal violet solution. After 2 washes with water (Milli Q), several microscopic fields were taken randomly, and the migrated cells were counted using ImageJ software.

#### 3.8.5. Tumor-Sphere Formation Assay

Three-dimensional or tumor-sphere culture is a recently introduced in vitro technique which maintains a physiological environment which closely resembles that of in vivo conditions [37]. This technique has now been widely used for the screening of anticancer moieties [41]. In vitro tumor-sphere formation assay was performed following a reported protocol [43]. Briefly, approximately 3 × 10^3^ cells were resuspended in a poly-2-hydroxyethyl methacrylate coated 6-well plate containing a sphere-forming medium with or without tested compounds and incubated for one week. The number of tumor spheres were counted, and the diameter of each tumor sphere was measured.

### 3.9. Determination of the Absolute Sugar Configuration

The absolute configuration of the sugar moieties of compound **1** and **2** was determined through GLC-MS/MS analysis of the octylated sugar moiety, after hydrolysis, employing the methods described previously [44,45], with some modifications. For hydrolysis, 0.5 mg of sample and 1 mL 2 M trifluoroacetic acid (TFA) were combined in a glass vial. The mixture was heated to 120 °C for 1 h. Afterwards, the mixture was washed three times, adding 5 mL water each time, by evaporating to dryness under reduced pressure. For octylation, 1 mL of (*R*)-(−)-2-octanol, and one drop of TFA (conc.) were added to the mixture. The sample was kept at 120 °C for 12 h. Subsequently, the sample was transferred to a separation funnel incorporating 5 mL of methanol with a few drops of water and separated three times with 5 mL *n*-hexane each time to remove excess octanol. The methanol fraction was evaporated under reduced pressure. For acetylation, the sample was heated in a vial at 100 °C for 20 min after adding 0.5 mL anhydrous acetic anhydride and 0.5 mL anhydrous pyridine.

After cooling the mixture at room temperature, 10 mL of water, 1 mL 0.1 M H_2_SO_4_, and 1 mL CH_2_Cl_2_ were added and the mixture was shaken vigorously. The CH_2_Cl_2_ layer was used for GLC–MS/MS analysis. For comparison with standard, 0.5 mg of d-glucose and 0.5 mg of l-glucose each were separately treated following the same procedure. GLC-MS analysis was performed using the column TG-5 SILMS (Thermo Scientific, 15 m × 0.25 mm × 0.25 μm) with the following settings: injection volume 1 μL; flow rate 1.2 mL/min; mobile phase helium; split ratio 1:10; ion source temperature 280 °C; injector temperature 290 °C; MS transfer line temperature 280 °C; scanning range for full scan: (*m*/*z*) 43–700; ionization mode EI; temperature gradient: 0 min 60 °C, 2 min 60 °C, 4.6 min 180 °C, 5.16 min 180 °C, 39.5 min 280 °C, and 41.13 min 280 °C.

The GLC-MS signals of the (*R*)-(–)-2-octanyl derivatives of standard d-glucose and the split off sugar moieties from compounds **1** and **2** had the same retention times (*t*_R_ = 32.08 and 33.16 min) and nearly identical MS spectra. In contrast, the signals obtained from the derivative of the standard l-glucose had significantly different retention times (*t*_R_ = 31.73 and 32.27 min).

### 3.10. Statistical Analysis

All experiments were repeated in triplicate, and results were expressed as mean ± standard deviation. The analysis of variance (one way ANOVA) was performed to assess statistically significant differences among the tested compounds. Differences in the mean values were assessed by the Tukey test at a significance level of *p* < 0.05 by using GraphPad Prism v. 6.0 (GraphPad Software Inc., San Diego, CA, USA).

## 4. Conclusions

In this study, three terpenoids, along with five previously isolated phenylpropanoids from *C. infortunatum*, revealed their antidiabetic, anticholinesterase, and anticancer potentials. Among the tested compounds, compound **6** was confirmed to have the best therapeutic potential against mammalian α-amylase compared to the reference standard acarbose. On the other hand, compounds **3** and **5** displayed moderate antiproliferative, antimetastatic, and antitumor properties against TNBC cell lines. In this view, the findings extended the chemical diversity of *C. infortunatum* and hold promise for identifying further potential nor-isoprenoid and phenylpropanoids as lead compounds against diabetes and TNBC.

## Figures and Tables

**Table 1:** 1D-^1^H NMR (600 MHz) spectroscopic data for compounds **1** and **2**.

Position	1 ^a^	1 ^b^	2 ^a^	2 ^b^	2 ^c^
	δ_H_ (*J* in Hz)	δ_H_ (*J* in Hz)	δ_H_ (*J* in Hz)	δ_H_ (*J* in Hz)	δ_H_ (*J* in Hz)
1	1.54, *td* (12.5, 6.5)	1.43, *td* (12.5, 6.5)	1.58, *td* (12.5, 6.5)	1.45, *m*	1.40, *m*
	3.46, *m*	3.31, *dd* (13.0, 6.5)	3.48, *m* ^d^	3.34, *m*	3.16, *m*
2	2.11, *dd* (18.5, 6.0)	2.00, *dd* (18, 6.0)	2.06, *m*	1.23, *m*	1.98, *m*
	2.32, *m*	2.20, *t* (10.0)	2.29, *m*		2.14, *m*
5	2.94, br *d* (15.5)	2.83, br *d* (15.5)	3.08, *m*	2.53, *m*	2.90, *m*
6	2.59, *dd* (16.5, 15.5)	2.94, *m* ^d^			2.39, *m*
	3.01, *dd* (17.0, 3.0)	2.50, *m* ^d^			2.50, *m*
14	7.47, *s*	7.42, *s*	7.41, *s*	7.41, *s*	7.35, *s*
15	2.67, *dd* (13.5, 7.0)	2.66, *dd* (13.5, 6.0)	2.67, *m* ^d^	2.67, *dd* (13.5, 6.0)	2.62, *dd* (13.5, 6.0)
	3.25, *dd* (13.5, 6.0)	2.92, *m* ^d^	3.27, *dd* (13.5, 6.5)	2.93, *dd* (13.5, 6.5)	2.93, *dd* (13.5, 6.5)
16	4.17, *dd* (13.0, 6.5)	3.97, *dd* (12.5, 6.0)	4.16, *dd* (13.0, 6.5)	3.97, *dd* (12.5, 6.0)	3.99, *dd* (12.5, 6.0)
17	1.10, *d* (6.0)	1.00, *d* (6.0)	1.00, *d* (6.0)	1.00, *d* (6.0)	0.97, *d* (6.0)
18	1.78, *s*	1.70, *s*	1.75, *s*	1.70, *s*	1.60, *s*
19	4.08, *d* (12.0)	3.85, *d* (12.0)			
	4.28, *d* (12.0)	4.13, *d* (12.0)			
20	1.28, *s*	1.18, *s*	1.32, *s*	1.21, *s*	1.10, *s*
1′	4.72, *d* (8.0)	4.64, *d* (8.0)	4.87, *d* (8.0)	4.74, *d* (8.0)	4.74, *m* ^d^
2′	3.88, *dd* (9.0, 8.0)	3.70, *m* ^d^	3.88, *dd* (17.0, 8.0)	3.71, *m* ^d^	3.79, *m* ^d^
3′	3.41, *m* ^d^	3.50, *m* ^d^	3.42, *m* ^d^	3.51, *m* ^d^	3.35, *m* ^d^
4′	3.47, *m* ^d^	3.23, *m* ^d^	3.48, *m* ^d^	3.23, *m* ^d^	3.39, *m* ^d^
5′	3.37, *m* ^d^	3.10, *m* ^d^	3.39, *m* ^d^	3.11, *m* ^d^	3.26, *m* ^d^
6′	3.67, *m* ^d^	3.42, *dd* (12.0, 6.0)	3.68, *dd* (12.0, 6.0)	3.44, *dd* (12.0, 6.0)	3.49, *dd* (12.0, 6.0)
	3.83, *m* ^d^	3.66, *m d*	3.82, *dd* (6.0, 1.5)	3.65, *m* ^d^	3.62, *m* ^d^
1″	4.87, *d* (8.0)	4.75, *d* (8.0)	4.73, *d* (8.0)	4.64, *d* (8.0)	4.71, *m* ^d^
2″	3.38, *m* ^d^	3.12, *m* ^d^	3.40, *m* ^d^	3.12, *m* ^d^	3.25, *m* ^d^
3″	3.66, *m* ^d^	3.20, *m* ^d^	3.67, *m* ^d^	3.21, *m* ^d^	3.58, *m* ^d^
4″	3.30, *m* ^d^	3.24, *m* ^d^	3.36, *m* ^d^	3.23, *m* ^d^	3.28, *m* ^d^
5″	3.36, *m* ^d^	3.18, *m* ^d^	3.31, *m* ^d^	3.19, *m* ^d^	3.21, *m* ^d^
6″	3.72, *dd* (12.0, 5.5)	3.47, *m* ^d^	3.72, *dd* (12.0, 5.0)	3.48, *m* ^d^	3.59, *m* ^d^
	3.83, *m* ^d^	3.68, *m* ^d^	3.84, *dd* (6.0, 2.0)	3.68, *m* ^d^	3.64, *m* ^d^

^a^ Spectra were referenced to solvent residual and solvent signals of CD_3_OD at 3.31 ppm (^1^H NMR, 600 MHz). ^b^ Spectra were referenced to solvent residual and solvent signals of (CD_3_)_2_SO at 2.50 ppm (^1^H NMR, 600 MHz). ^c^ Spectra were referenced to solvent residual D_2_O at 4.59 ppm (^1^H NMR, 600 MHz). ^d^ Overlapping.

**Table 2:** 1D-^13^C NMR (150 MHz) spectroscopic data for compounds **1** and **2**.

Position	1 ^a^	1 ^b^	2 ^a^	2 ^b^	2 ^c^
	δ_C_, Type	δ_C_, Type	δ_C_, Type	δ_C_, Type	δ_C_, Type
1	32.7, CH_2_	31.1, CH_2_	32.5, CH_2_	30.7, CH_2_	32.3, CH_2_
2	31.1, CH_2_	29.7, CH_2_	29.7, CH_2_	29.0, CH_2_	29.6, CH_2_
3	130.2, C	129.2, C	130.5, C	129.4, C	131.2, C
4	132.5, C	129.9, C	132.5, C	129.6, C	133.3, C
5	44.4, CH	42.7, CH	43.2, CH	40.4, CH	42.5, CH
6	38.1, CH_2_	36.6, CH_2_	38.6, CH_2_	36.9, CH_2_	38.9, CH_2_
7	200.9, C	197.8, C	200.3, C	197.0, C	202.9, C
8	129.5, C	128.3, C	128.4, C	128.4, C	130.4, C
9	139.7, C	137.2, C	139.6, C	136.8, C	140.5, C
10	39.0, C	37.3, C	39.1, C	37.5, C	38.7, C
11	149.4, C	147.8, C	149.3, C	147.8, C	149.1, C
12	150.2, C	148.4, C	150.1, C	148.4, C	150.4, C
13	133.8, C	131.4, C	134.6, C	131.6, C	133.0, C
14	122.5, CH	120.7, CH	122.7, CH	121.0, CH	123.4, CH
15	41.1, CH_2_	39.2, CH_2_	41.1, CH_2_	39.2, CH_2_	40.0, CH_2_
16	68.1, CH	65.5, CH	68.1, CH	65.5, CH	68.6, CH
17	22.7, CH_3_	23.3, CH_3_	22.6, CH_3_	23.3, CH_3_	23.4, CH_3_
18	19.0, CH_3_	18.8, CH_3_	20.5, CH_3_	20.1, CH_3_	21.2, CH_3_
19	59.2, CH_2_	57.5, CH_2_	----	166.2, COOH	170.7, COOH
20	15.7, CH_3_	15.2, CH_3_	16.1, CH_3_	15.3, CH_3_	16.8, CH_3_
1′	105.5, CH	103.6, CH	105.3, CH	103.6, CH	104.8, CH
2′	82.7, CH	80.8, CH	82.7, CH	80.7, CH	82.0, CH
3′	77.8, CH	76.1, CH	77.8, CH	76.1, CH	77.1, CH
4′	70.8, CH	69.5, CH	70.8, CH	69.5, CH	70.4, CH
5′	71.4, CH	69.9, CH	71.4, CH	69.9, CH	71.0, CH
6′	62.6, CH_2_	61.1, CH_2_	62.6, CH_2_	61.1, CH_2_	62.1, CH_2_
1″	105.4, CH	103.6, CH	105.5, CH	103.7, CH	104.9, CH
2″	75.7, CH	74.1, CH	75.7, CH	74.1, CH	75.2, CH
3″	77.9, CH	76.2, CH	77.9, CH	76.2, CH	77.2, CH
4″	78.6, CH	77.5, CH	78.6, CH	77.5, CH	77.9, CH
5″	78.5, CH	77.4, CH	78.6, CH	77.4, CH	77.8, CH
6″	62.3, CH_2_	60.9, CH_2_	62.2, CH_2_	60.8, CH_2_	61.7, CH_2_

^a^ Spectra were referenced to solvent residual and solvent signals of CD_3_OD at 49.0 ppm (^13^C NMR, 150 MHz). ^b^ Spectra were referenced to solvent residual and solvent signals of (CD_3_)_2_SO at 39.52 ppm (^13^C NMR, 150 MHz). ^c^ Spectra were referenced to solvent residual and solvent signals of D_2_O.

**Table 3:** Comparison of partial NMR data of **1** and **2** with known compounds ^a^.

Position	Szemaoenoid A		Szemaoenoid C		E		Compound 1		Compound 2	
	δ_C_	δ_H_	δ_C_	δ_H_	δ_C_	δ_H_	δ_C_	δ_H_	δ_C_	δ_H_
15	40.9	2.71, 3.20	33.6	2.87, 3.17	40.4	2.70, 2.82	41.1	2.67, 3.25	41.1	2.67, 3.27
16	68.3	4.10	68.3	4.15	68.7	4.04	68.1	4.17	68.1	4.16
17	22.8	1.12	22.9	1.12	23.3	1.15	22.7	1.10	22.6	1.00

^a^ Spectra of all compounds were measured in CD_3_OD. E = (5*R*,10*S*,16*R*)-11,16-dihydroxy-12 methoxy-17(15→16)-abeoabieta-8,11,13-trien-3,7-dione.

**Table 4:** Enzyme inhibition activity of three terpenoids (**1**–**3**) and five phenylpropanoids (**4**–**8**) against α-amylase, α-glucosidase, AChE, and BChE in comparison with standard acarbose and galanthamine.

Compound	IC_50_ (µM)			
	α-Amylase	α-Glucosidase	AChE	BChE
**1**	18.5 ± 0.6 ^b^	24.6 ± 0.2 ^a^	191 ± 10.2 ^a^	>1000
**2**	64.6 ± 7.1 ^c^	78.3 ± 3 ^a^	139 ± 7.2 ^a^	>1000
**3**	284 ± 13.2 ^d^	>1000	>1000	>1000
**4**	13.0 ± 1.3 ^a,b^	>1000	>1000	>1000
**5**	24.9 ± 0.4 ^b^	96 ± 10.5 ^a^	160 ± 12.2 ^a^	>1000
**6**	3.4 ± 0.2 ^a^	55.8 ± 0.2 ^a^	>1000	>1000
**7**	221 ± 24.5 ^d^	>1000	>1000	>1000
**8**	19.8 ± 0.50 ^b^	>1000	178 ± 11.3 ^a^	>1000
acarbose	5.9 ± 0.1	665 ± 42		
galanthamine			2.9 ± 0.4	22.5 ± 1.9

Values are expressed as mean ± SD (*n* = 3). Different superscript letters correspond to values considered statistically different (*p* ≤ 0.05).

**Table 5:** Cytotoxic activity of the tested compounds at a half maximal inhibitory concentration (IC_50_) in the TNBC cell lines Hs578T and MDA-MB-231.

Compounds	IC_50_ (μM)	
	Hs578T	MDA-MB-231
**1**	128 ± 2.2	122 ± 1.2
**3**	94.7 ± 1.3	118 ± 3.3
**4**	210 ± 5.1	199 ± 3.1
**5**	85.3 ± 2.4	96.5 ± 1.5

## Data Availability

Not Applicable.

## Supplementary Materials

The following are available online. Supplementary data associated with this article are ^1^H, ^13^C, COSY, HSQC, and HMBC NMR spectra of compounds **1** and **2** in CD_3_OD, DMSO-*d_6_*_,_ and D_2_O at 600 MHz; HR-ESI-MS in acetone for compounds **1** and **2**; ^1^H and ^13^C NMR data in CD_3_OD, and DMSO-*d_6_* at 600 MHz of known compound **3**.

## Sample Availability

Samples are not available from the authors as they were consumed during measurements and bioassays.
