# Supplementary material for: Bioactive Abietane-Type Diterpenoid Glycosides from Leaves of Clerodendrum infortunatum (Lamiaceae)
Source: Molecules. 2021 Jul 6;26(14):4121. doi: 10.3390/molecules26144121 (PMC8306933; doi:10.3390/molecules26144121)
Supplement: Supplementary file 1 [file molecules-26-04121-s001.zip › molecules-1261738-supplementary.pdf]

# Supplementary Materials

## Bioactive abietane-type diterpenoid glycosides from leaves of *Clerodendrum infortunatum* (Lamiaceae)

**Md. Josim Uddin[1,2], Daniela Russo[3,4], Md. Anwarul Haque[5,6], Serhat Sezai Çiçek[1], Frank D. Sönnichsen[7], Luigi Milella[3], and Christian Zidorn[1,*]**

1 Pharmazeutisches Institut, Abteilung Pharmazeutische Biologie, Christian-Albrechts-Universität zu Kiel, Gutenbergstrasse 76, 24118 Kiel, Germany; juddin@pharmazie.uni-kiel.de; scicek@pharmazie.uni-kiel.de

2 Department of Pharmacy, International Islamic University Chittagong, Chittagong-4318, Bangladesh

3 Department of Science, University of Basilicata, Viale dell' Ateneo Lucano 10, 85100 Potenza, Italy; daiela.russo@unibas.it; luigi.milella@unibas.it

4 Spinoff BioActiPlant s.r.l., Viale dell' Ateneo Lucano 10, 85100 Potenza, Italy

5 Department of Experimental Pathology, Graduate School of Comprehensive Human Sciences, University of Tsukuba, Ibaraki 305-8575, Japan; a.haque5314@gmail.com

6 Department of Pharmacy, University of Rajshahi, Rajshahi-6205, Bangladesh

7 Otto Diels Institute for Organic Chemistry, University of Kiel, Otto-Hahn-Platz 4, 24118 Kiel, Germany; fsoennichsen@oc.uni-kiel.de

**Corresponding Author**

*Christian Zidorn: Pharmazeutisches Institut, Abteilung Pharmazeutische Biologie,

Christian-Albrechts-Universität zu Kiel, Gutenbergstraße 76, 24118 Kiel, Germany. Tel.:

+49-431-880-1139. E-mail: czidorn@pharmazie.uni-kiel.de

## List of contents


| Content | Page |
|---|---|
| Figure S1-A. 1D-$^{1}$H NMR (600 MHz) spectrum of compound **1** in Methanol-$d_4$. | 3 |
| Figure S1-B. 1D-$^{1}$H NMR (600 MHz) spectrum of compound **1** in DMSO-$d_6$. | 4 |
| Figure S2-A. 1D-$^{13}$C NMR (150 MHz) spectrum of compound **1** in Methanol-$d_4$. | 5 |
| Figure S2-B. 1D-$^{13}$C NMR (150 MHz) spectrum of compound **1** in DMSO-$d_6$. | 6 |
| Figure S3-A. COSY spectrum of compound **1** in Methanol-$d_4$. | 7 |
| Figure S3-B. COSY spectrum of compound **1** in DMSO-$d_6$. | 8 |
| Figure S4-A. HSQC spectrum of compound **1** in Methanol-$d_4$. | 9 |
| Figure S4-B. HSQC spectrum of compound **1** in DMSO-$d_6$. | 10 |
| Figure S5-A. HMBC spectrum of compound **1** in Methanol-$d_4$. | 11 |
| Figure S5-B. HMBC spectrum of compound **1** in DMSO-$d_6$. | 12 |
| Figure S6. HR mass spectrum of compound **1** in methanol. | 13 |
| Figure S7-A. 1D-$^{1}$H NMR (600 MHz) spectrum of compound **2** in Methanol-$d_4$. | 14 |
| Figure S7-B. 1D-$^{1}$H NMR (600 MHz) spectrum of compound **2** in DMSO-$d_6$. | 15 |
| Figure S7-C. 1D-$^{1}$H NMR (600 MHz) spectrum of compound **2** in $D_2O$. | 16 |
| Figure S8-A. 1D-$^{13}$C NMR (150 MHz) spectrum of compound **2** in Methanol-$d_4$. | 17 |
| Figure S8-B. 1D-$^{13}$C NMR (150 MHz) spectrum of compound **2** in DMSO-$d_6$. | 18 |
| Figure S8-C. 1D-$^{13}$C NMR (150 MHz) spectrum of compound **2** in $D_2O$. | 19 |
| Figure S9-A. COSY spectrum of compound **2** in Methanol-$d_4$. | 20 |
| Figure S9-B. COSY spectrum of compound **2** in DMSO-$d_6$. | 21 |
| Figure S9-C. COSY spectrum of compound **2** in $D_2O$. | 22 |
| Figure S10-A. HSQC spectrum of compound **2** in Methanol-$d_4$. | 23 |
| Figure S10-B. HSQC spectrum of compound **2** in DMSO-$d_6$. | 24 |
| Figure S10-C. HSQC spectrum of compound **2** in $D_2O$. | 25 |
| Figure S11-A. HMBC spectrum of compound **2** in Methanol-$d_4$. | 26 |
| Figure S11-B. HMBC spectrum of compound **2** in DMSO-$d_6$. | 27 |
| Figure S11-C. HMBC spectrum of compound **2** in $D_2O$. | 28 |
| Figure S12. HR mass spectrum of compound **2** in methanol. | 29 |
| Figure S13. HR mass spectrum of compound **3** in methanol. | 30 |
| Figure S14. UHPLC ($\lambda$ = 330 nm) chromatogram of *n*-butanol fraction from leaves of *Clerodendrum infortunatum* | 31 |
| Table S1. 1D ($^{1}$H, 600 MHz and $^{13}$C, 150 MHz) NMR spectroscopic data for compound **3** in $CD_3OD$ and $(CD_3)_2SO$ | 32 |

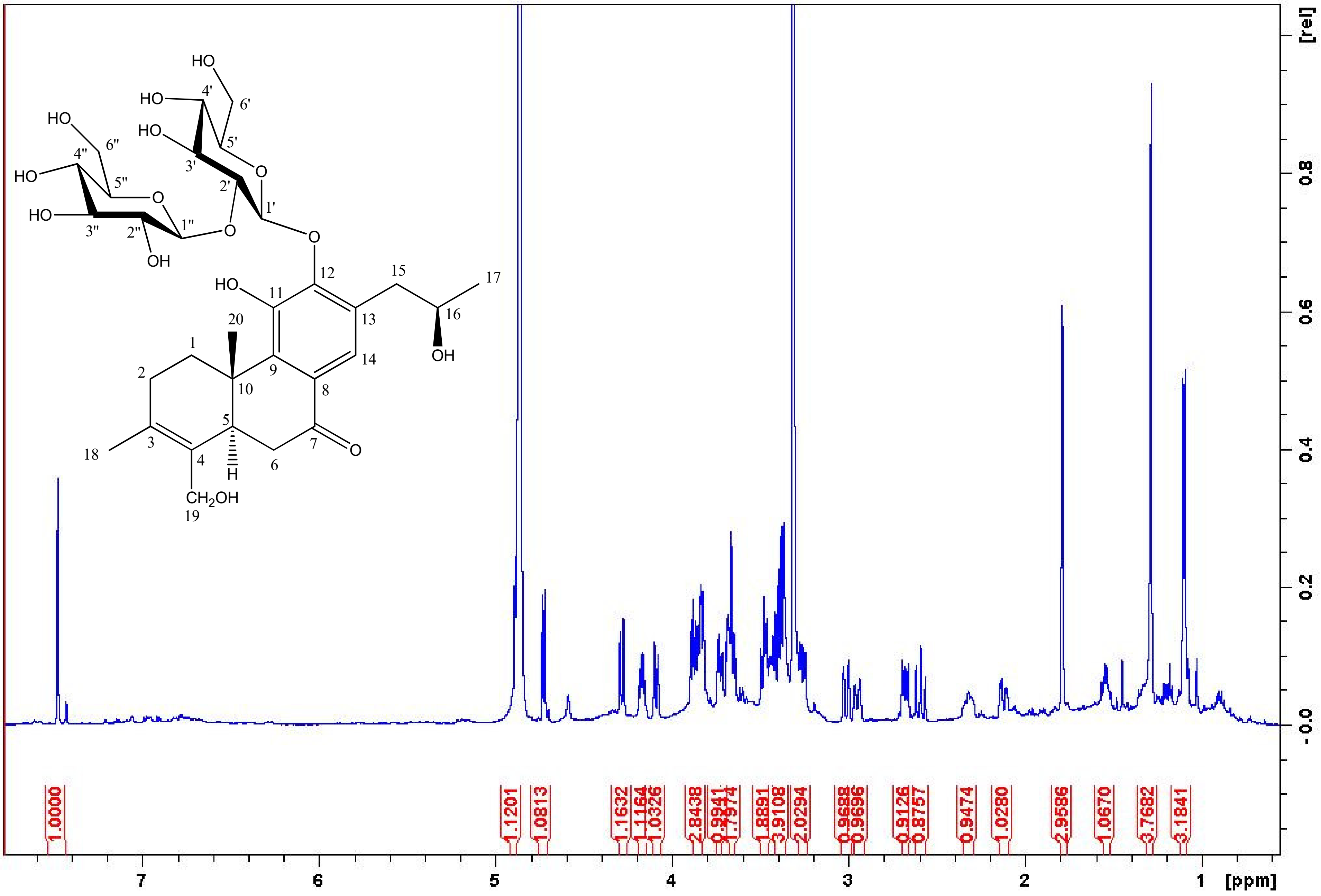

Figure S1-A. $^{1}$H NMR spectrum of compound **1** in Methanol-$d_4$.

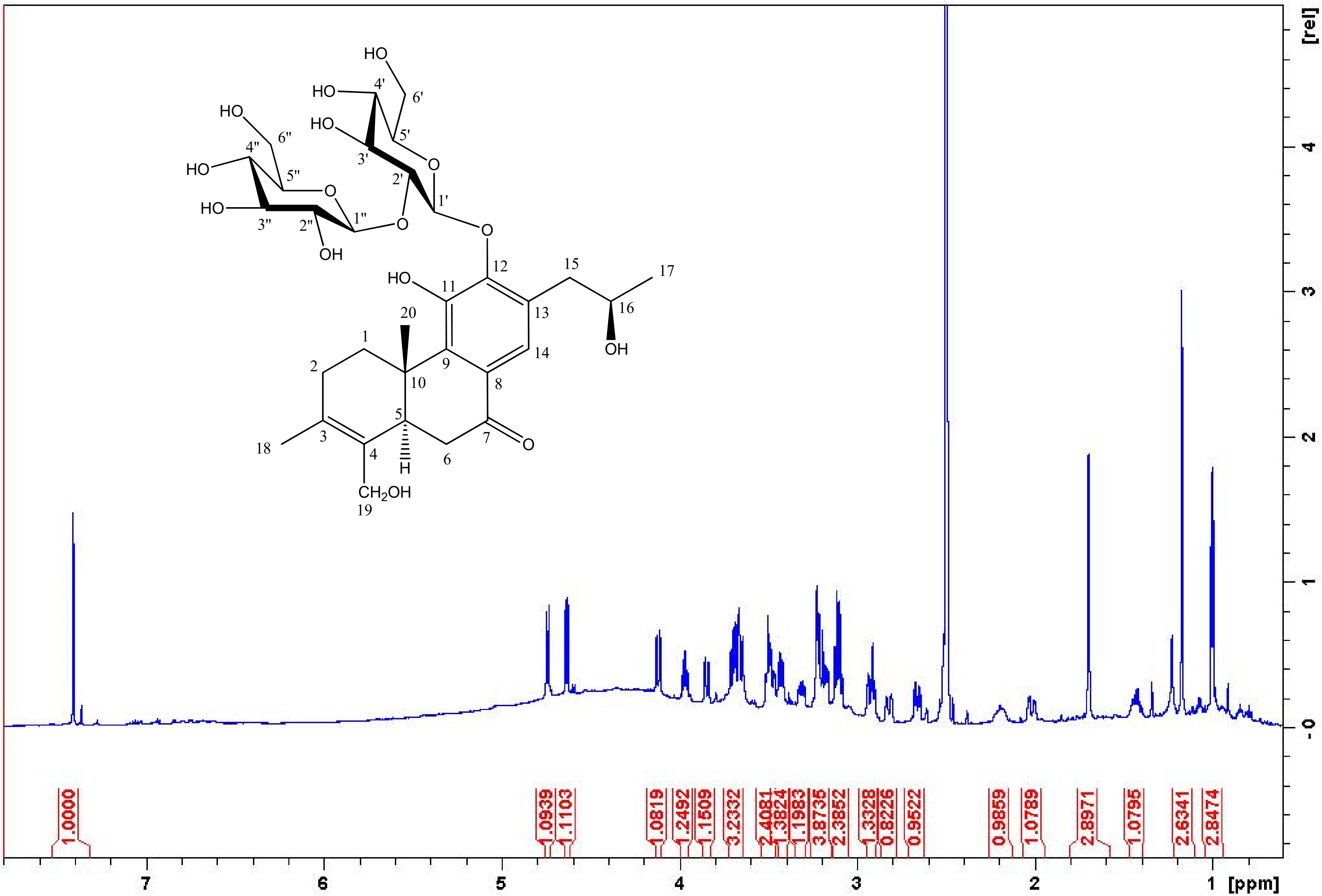


Figure S1-B. $^{1}$H NMR spectrum of compound **1** in DMSO-$d_6$

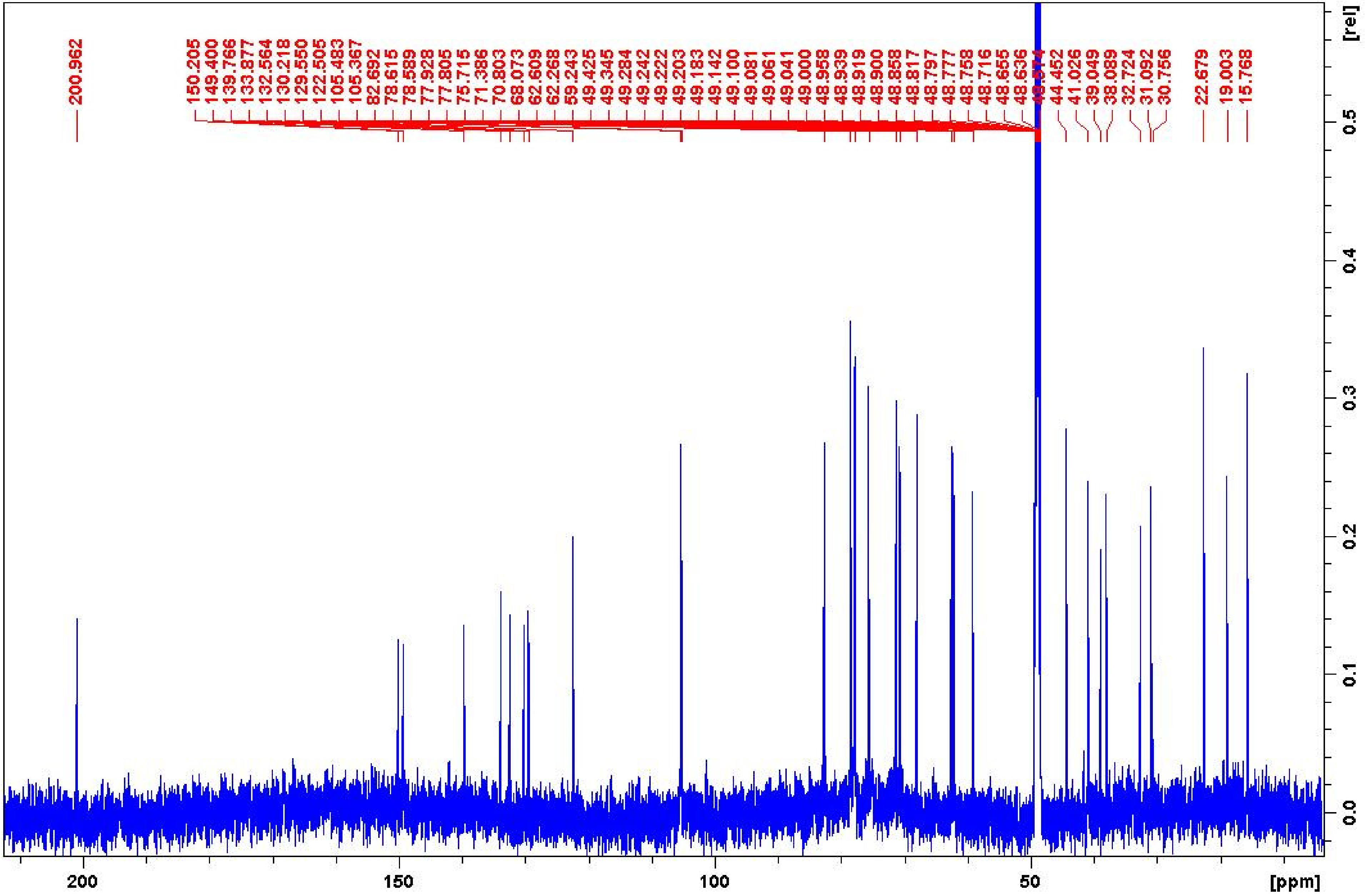

Figure S2-A. $^{13}C$ NMR spectrum of compound **1** in Methanol-$d_4$.

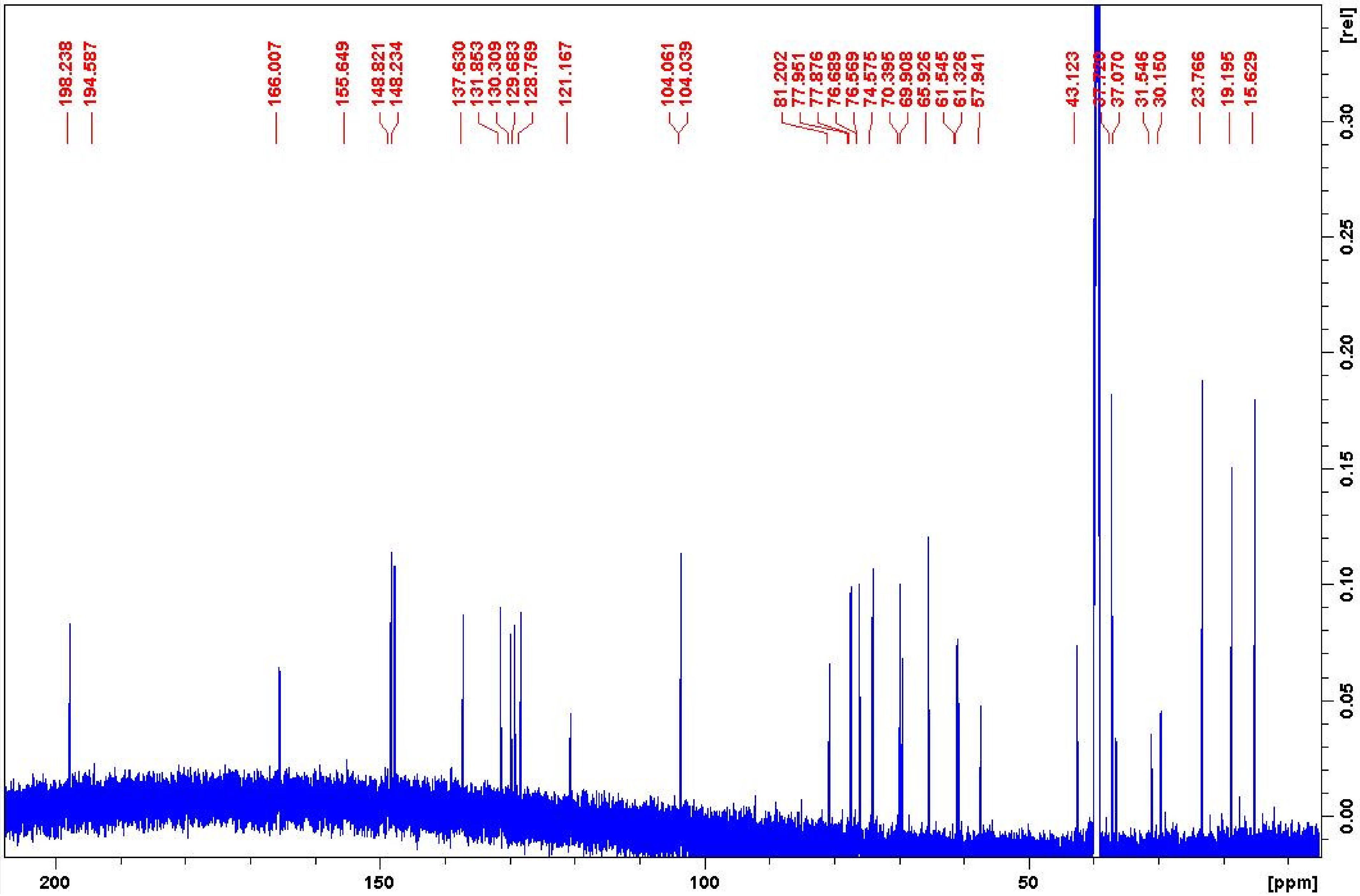


Figure S2-B. $^{13}C$ NMR spectrum of compound **1** in DMSO-$d_6$.

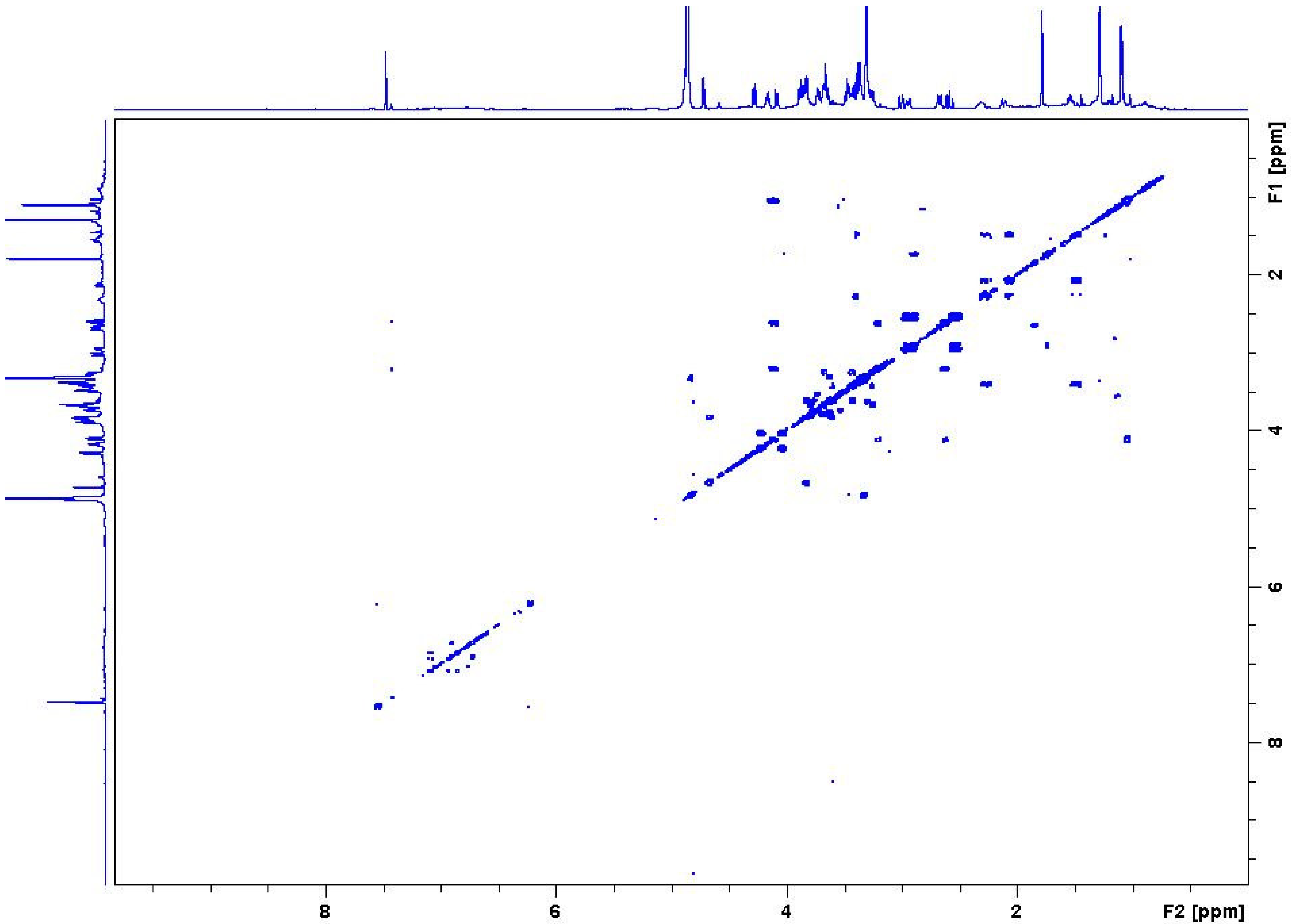


Figure S3-A. COSY spectrum of compound **1** in Methanol-$d_4$.

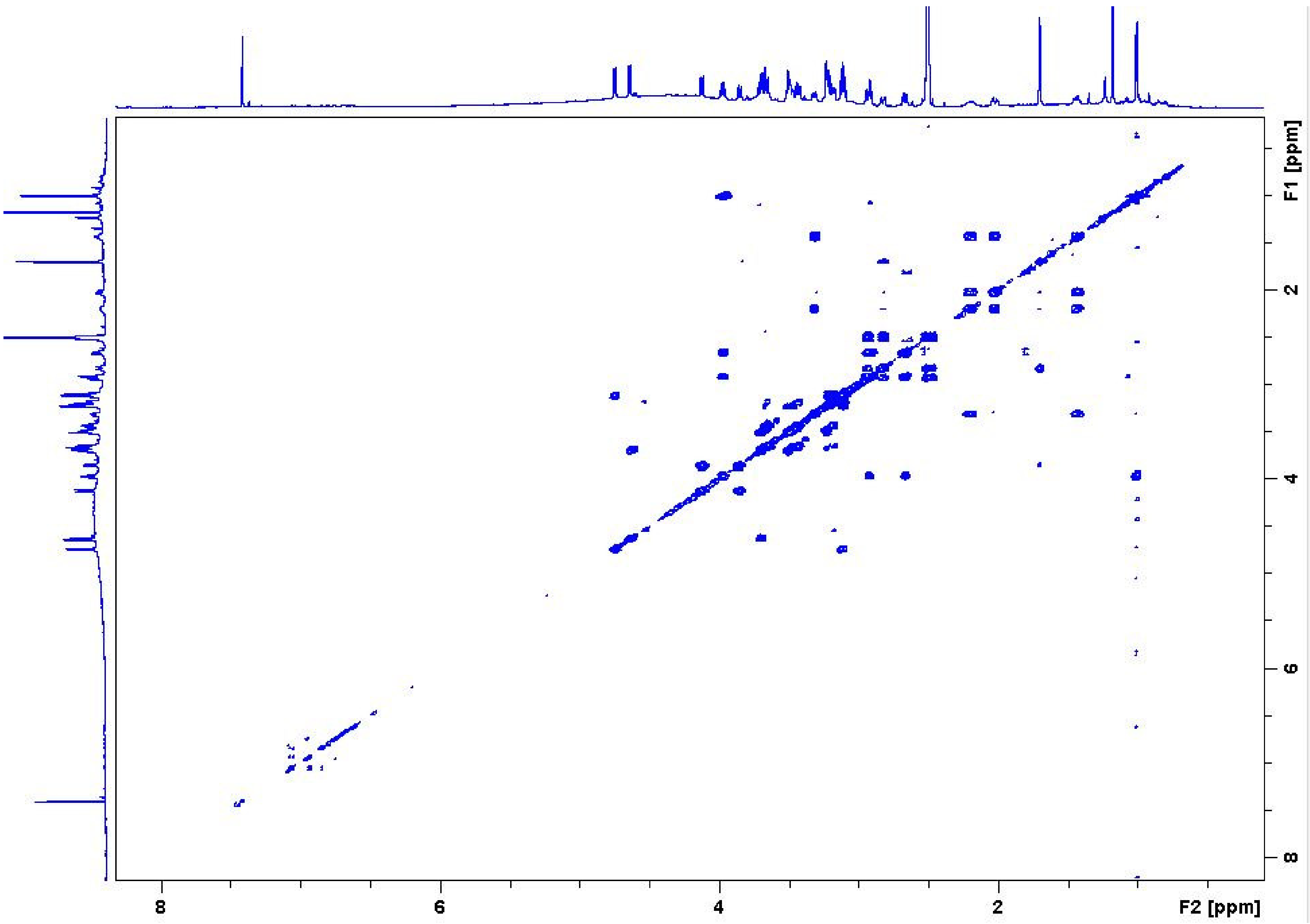


Figure S3-B. COSY spectrum of compound **1** in DMSO-$d_6$.

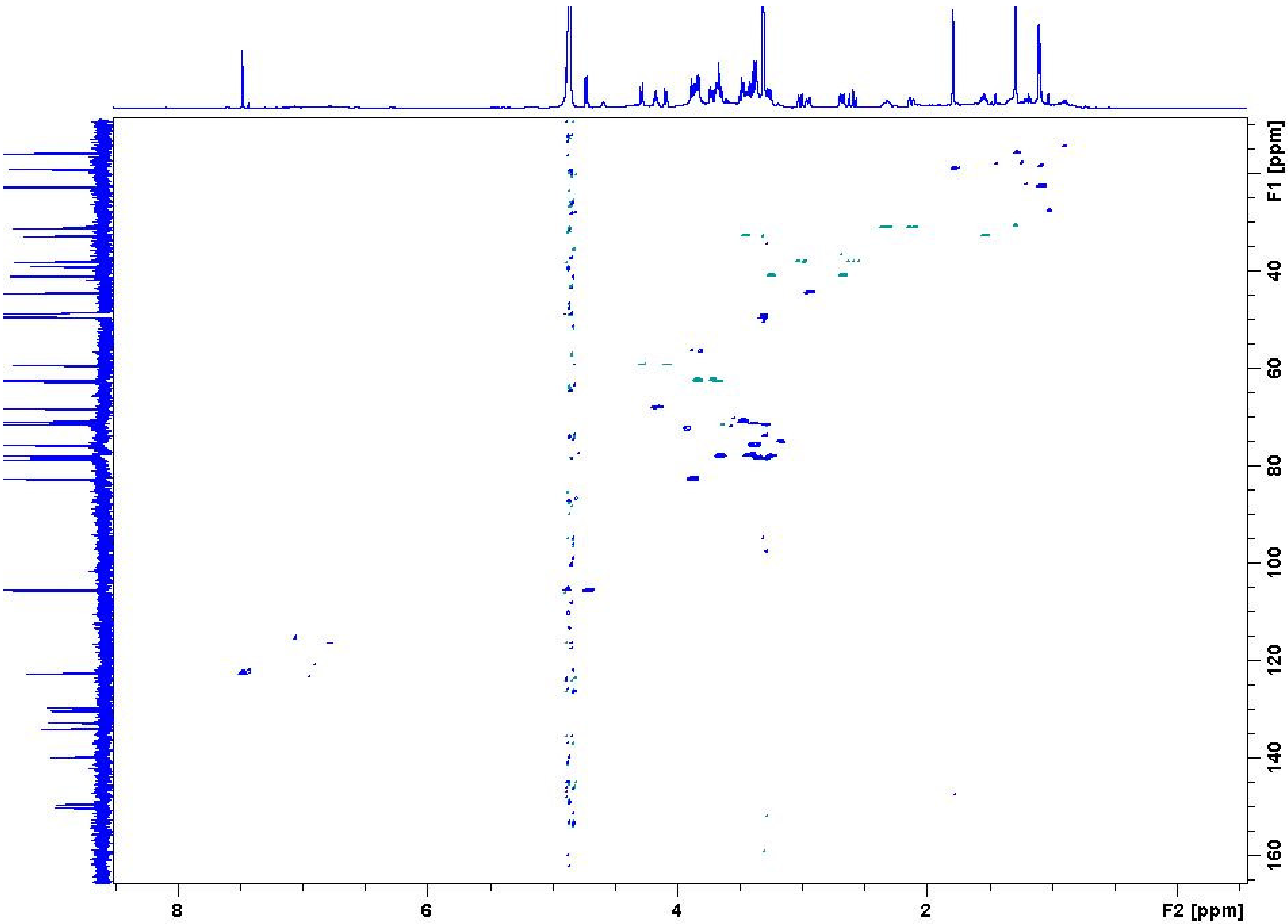


Figure S4-A. HSQC spectrum of compound **1** in Methanol-$d_4$.

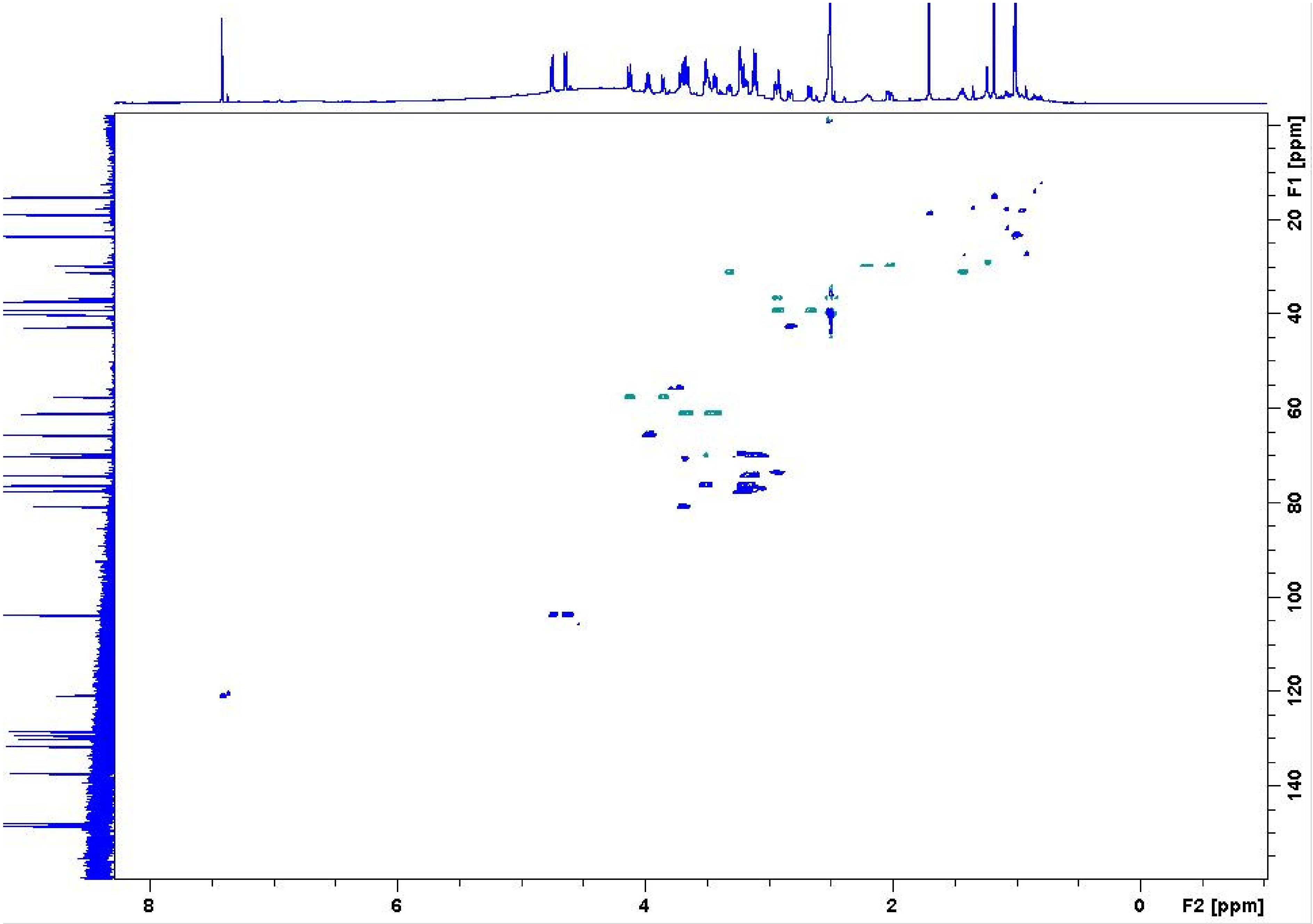


Figure S4-B. HSQC spectrum of compound **1** in DMSO-$d_6$.

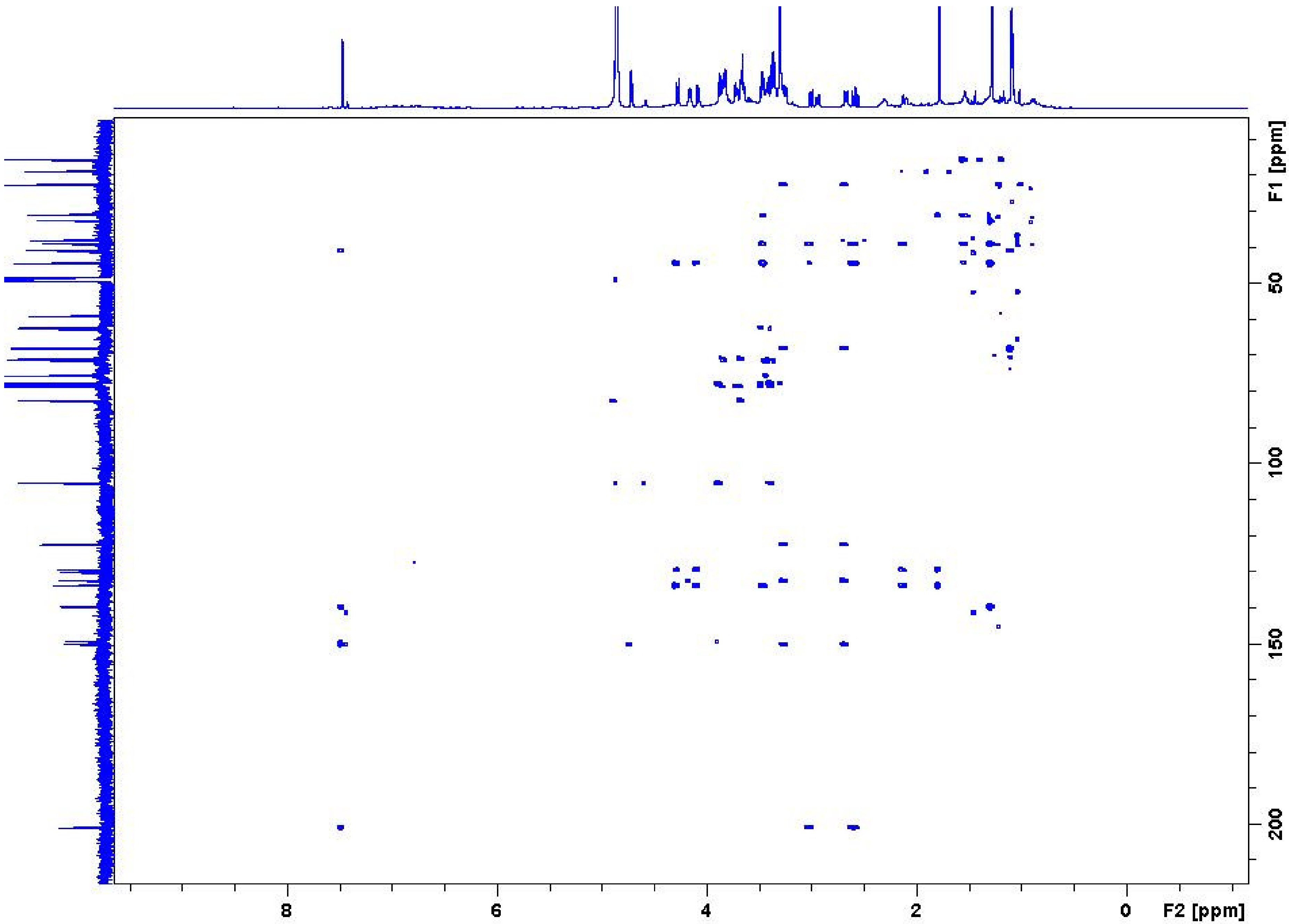


Figure S5-A. HMBC spectrum of compound **1** in Methanol-$d_4$.

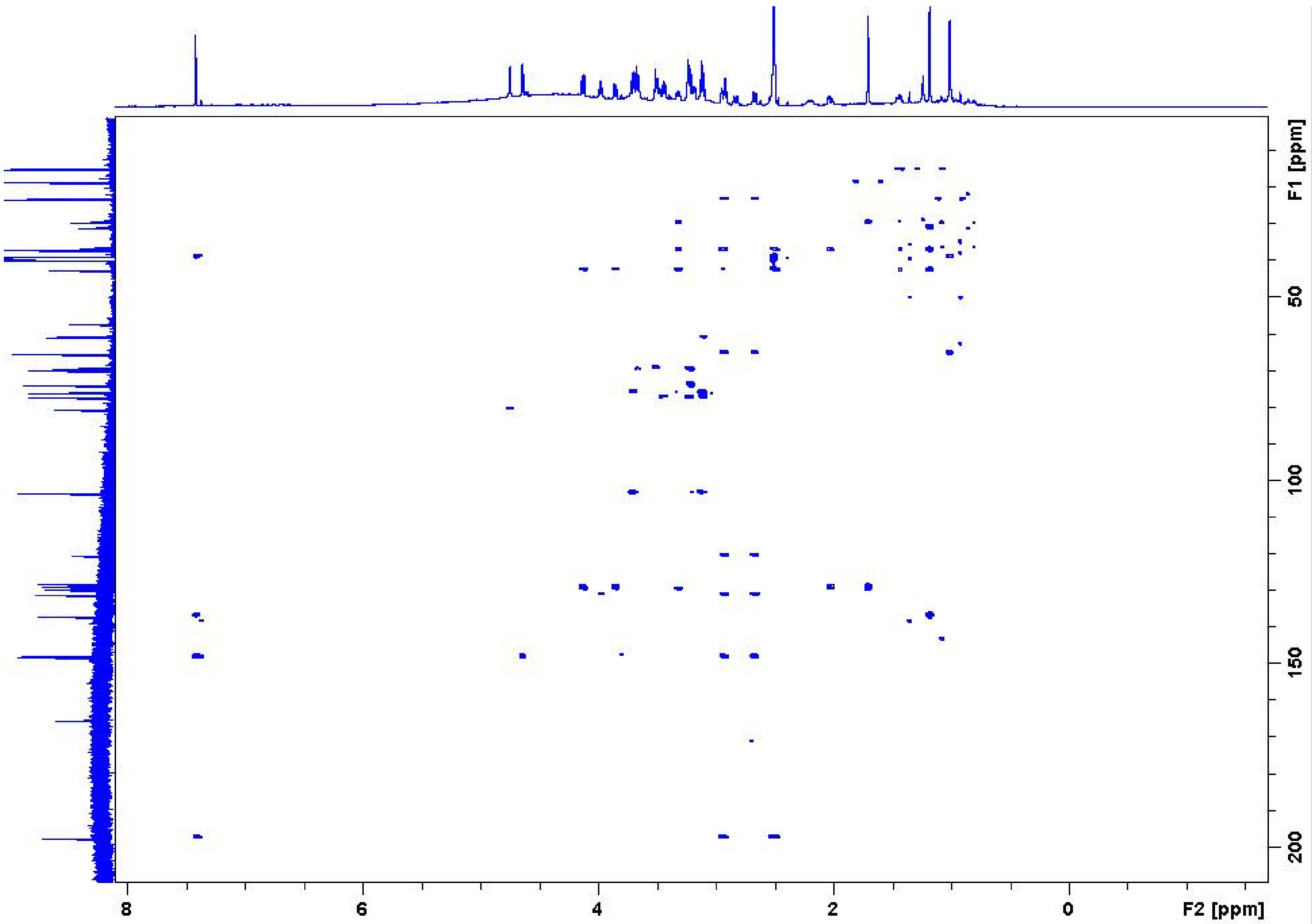


Figure S5-B. HMBC spectrum of compound **1** in DMSO-$d_6$.

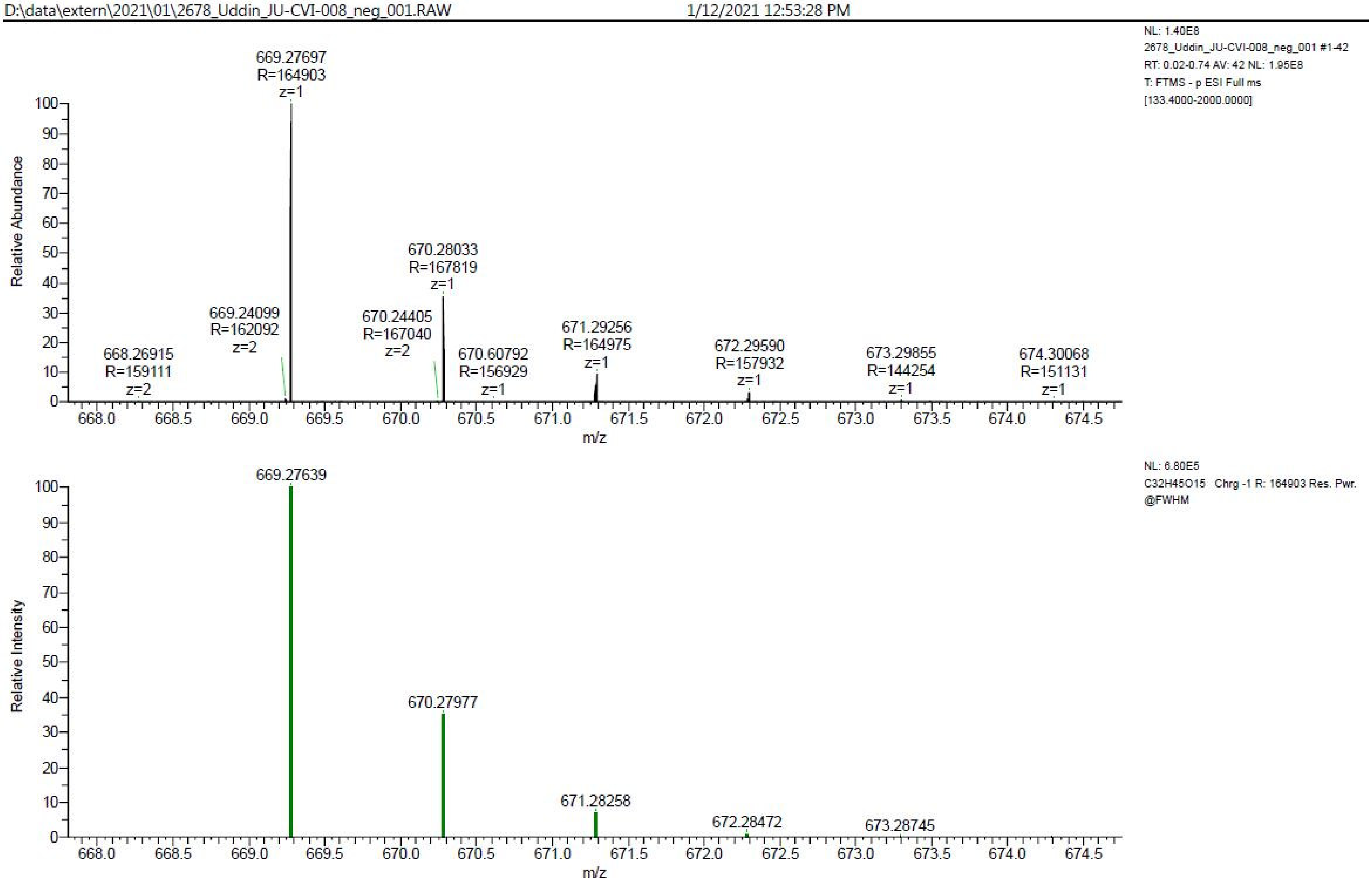


| Peak Mass | Display Form... | Combined Fit | RDB | Delta [ppm] | Theo. mass | Rank | Combined Sc... | # Matched Iso. | # Missed Iso. | MS Cov. [%] | Pattern Cov. [... | MSMS Match... |
|---|---|---|---|---|---|---|---|---|---|---|---|---|
| 669.27697 | $C_{32}H_{45}O_{15}$ | 94.3841840... | 10.50 | 0.85 | 669.27639 | 1 | 0 | 5 | 1 | 0 | 0 | (Collection) |

Figure S6. HR mass spectrum of compound **1** in methanol.

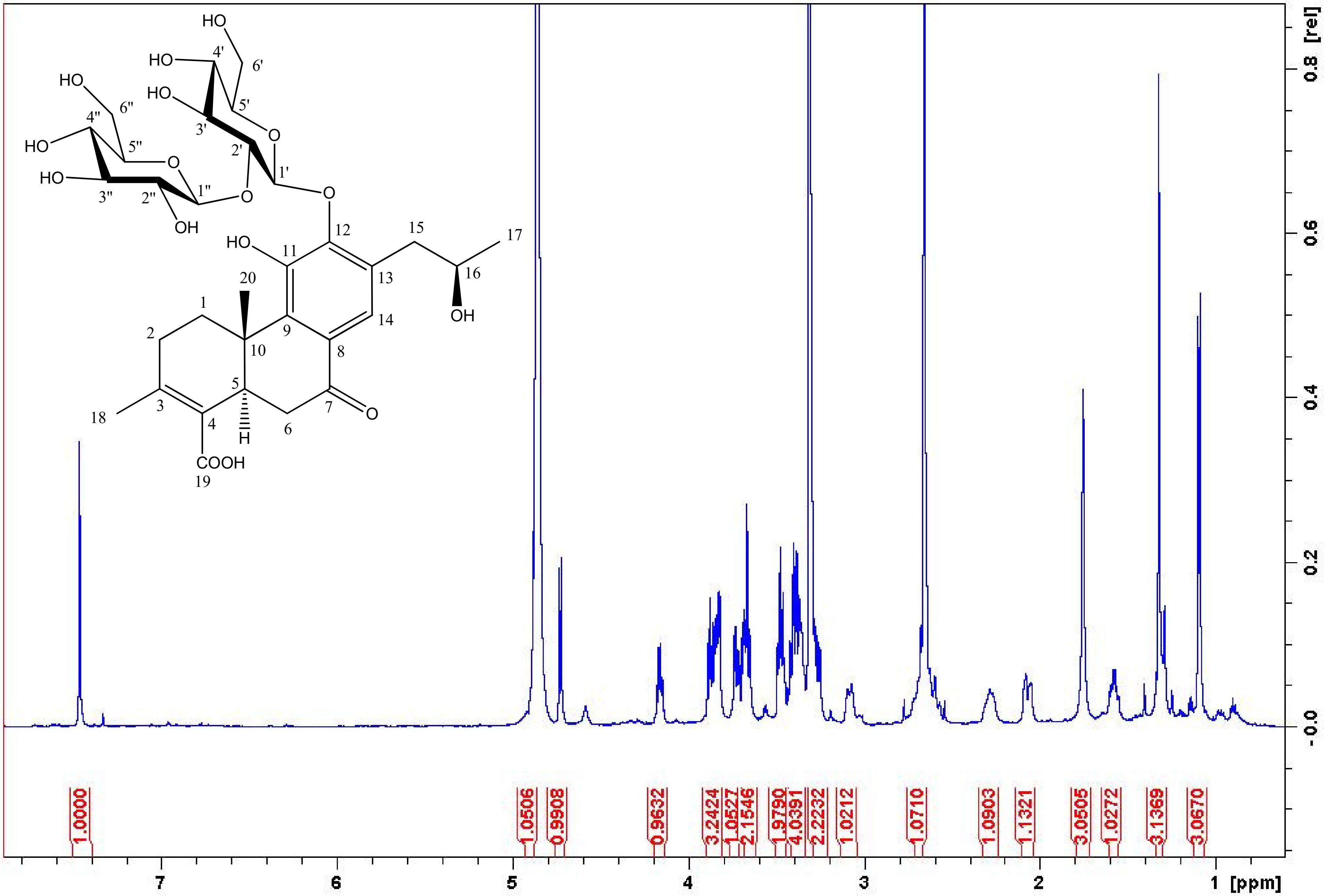


Figure S7-A. $^{1}$H NMR spectrum of compound **2** in Methanol-$d_4$.

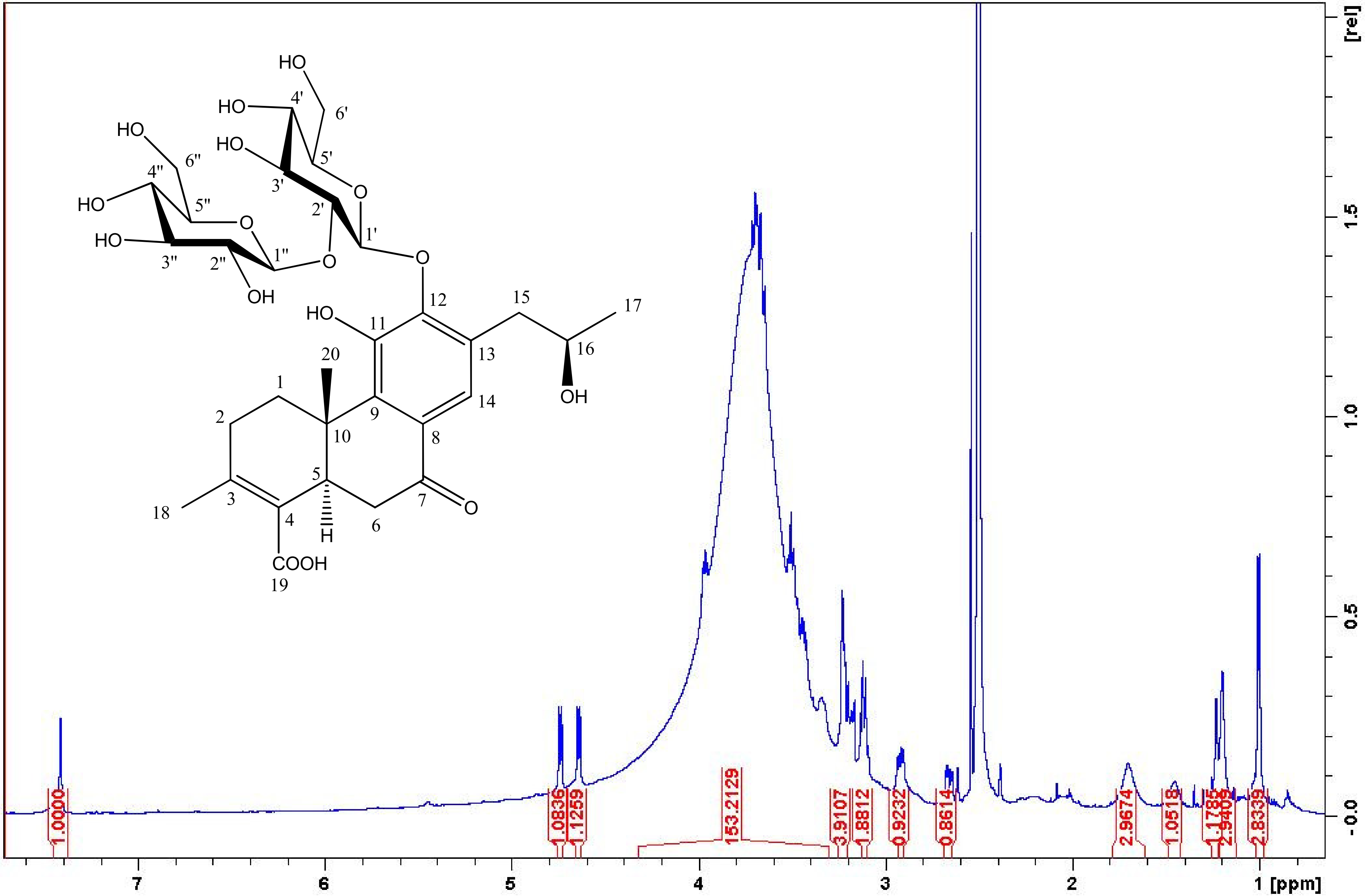


Figure S7-B. $^{1}H$ NMR spectrum of compound **2** in DMSO-$d_6$.

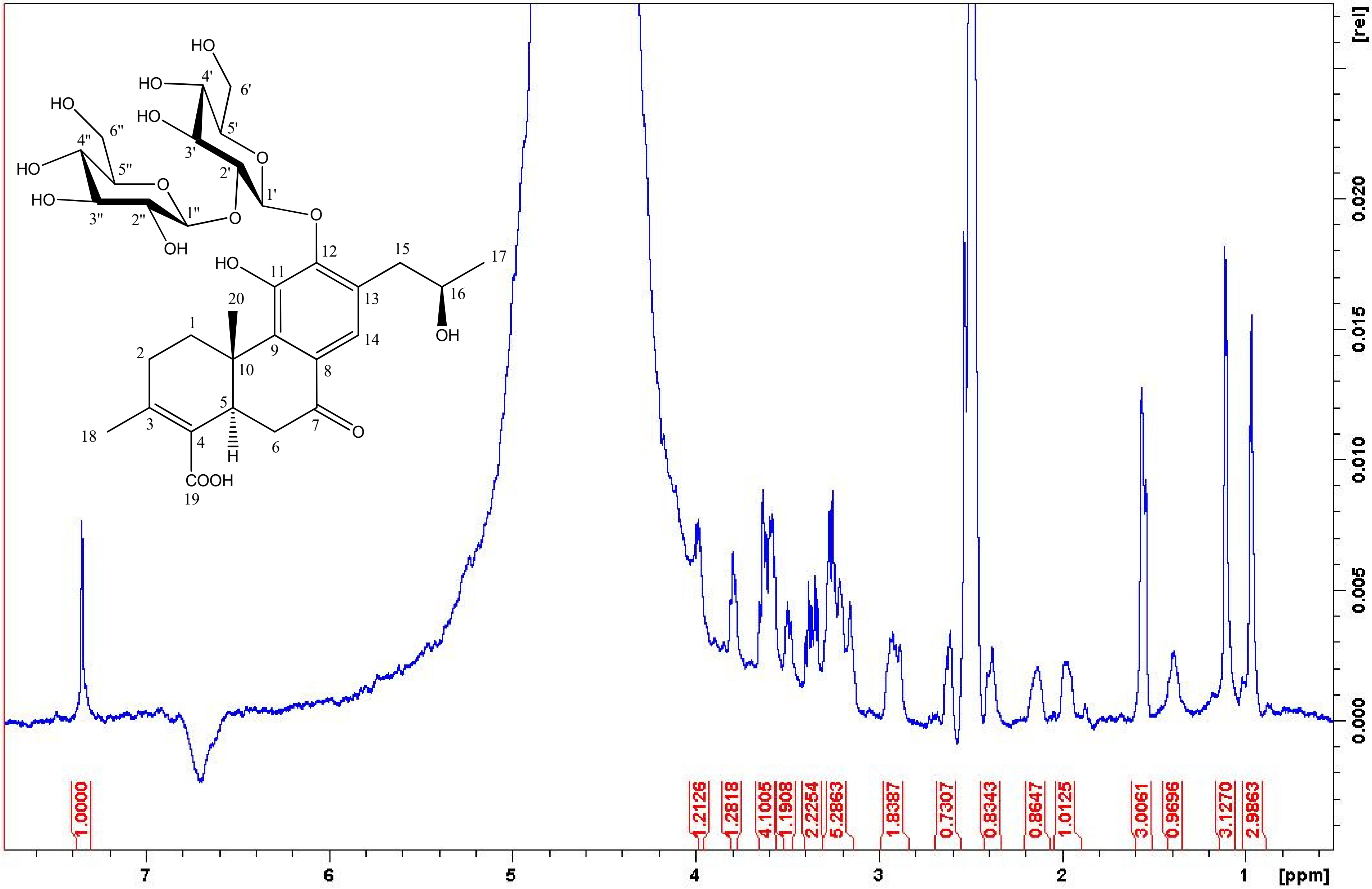


Figure S7-C. $^{1}$H NMR spectrum of compound **2** in $D_2O$.

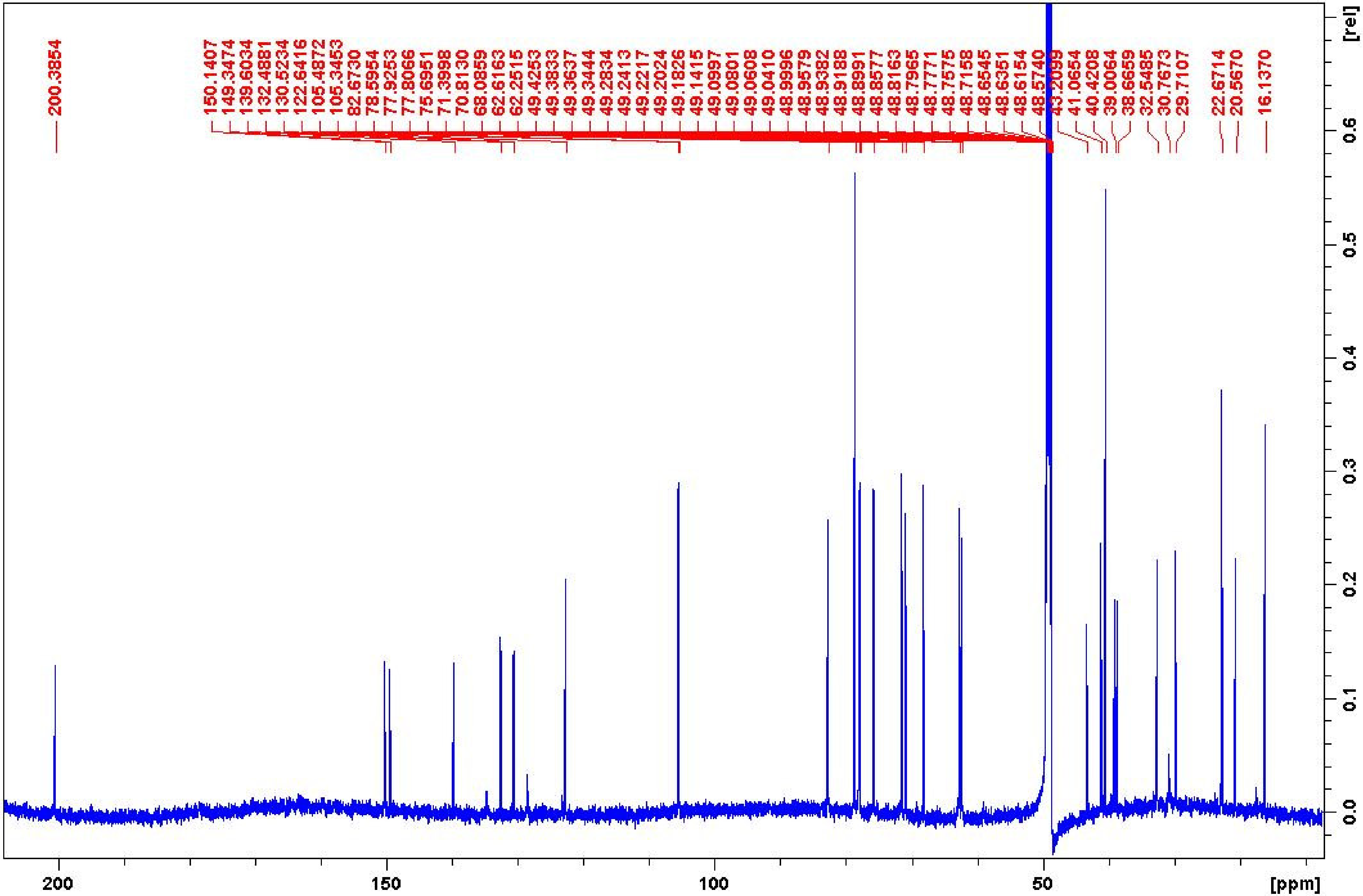

Figure S8-A. $^{13}C$ NMR spectrum of compound **2** in Methanol-$d_4$.

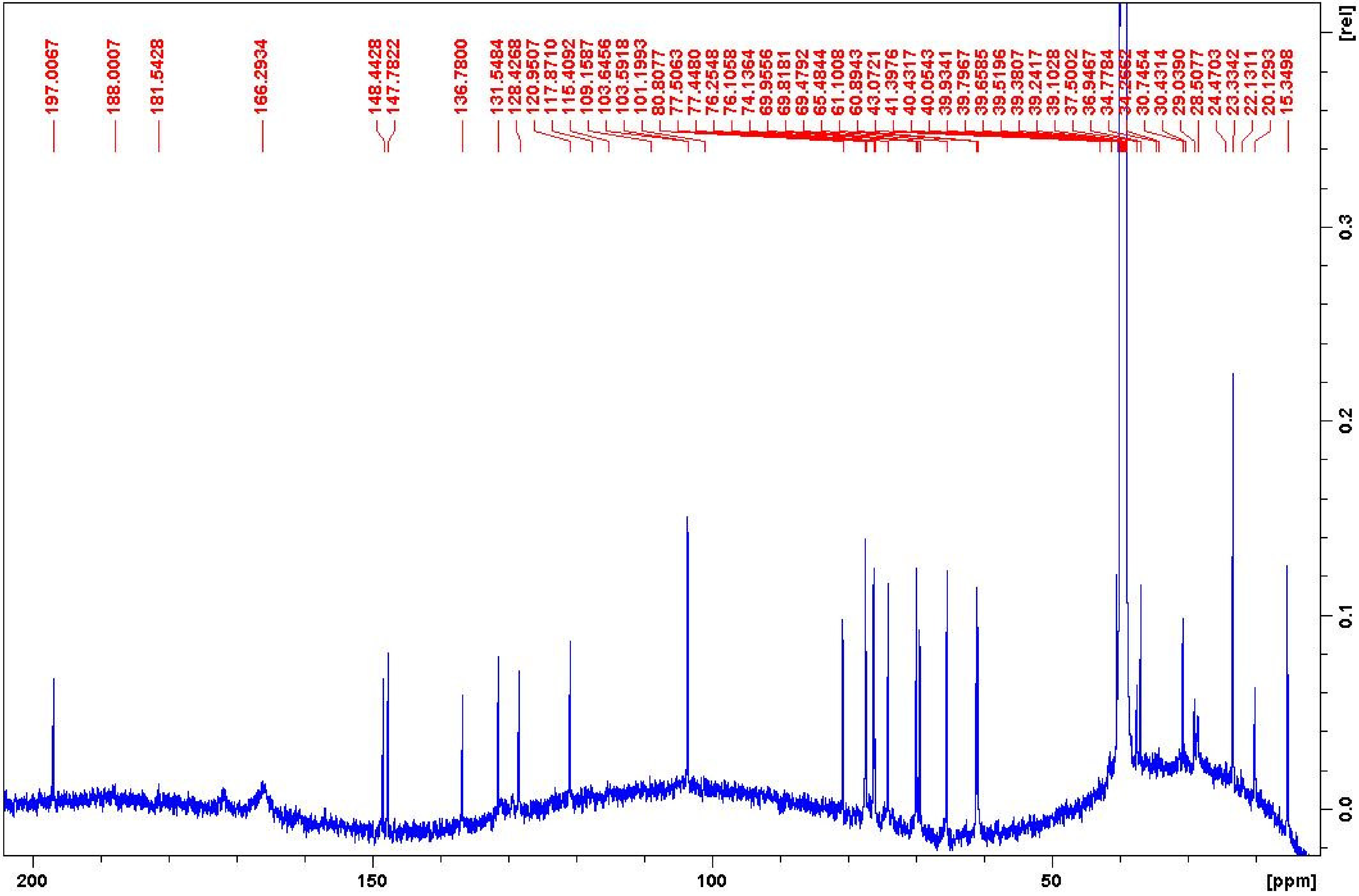


Figure S8-B. $^{13}C$ NMR spectrum of compound **2** in DMSO-$d_6$.

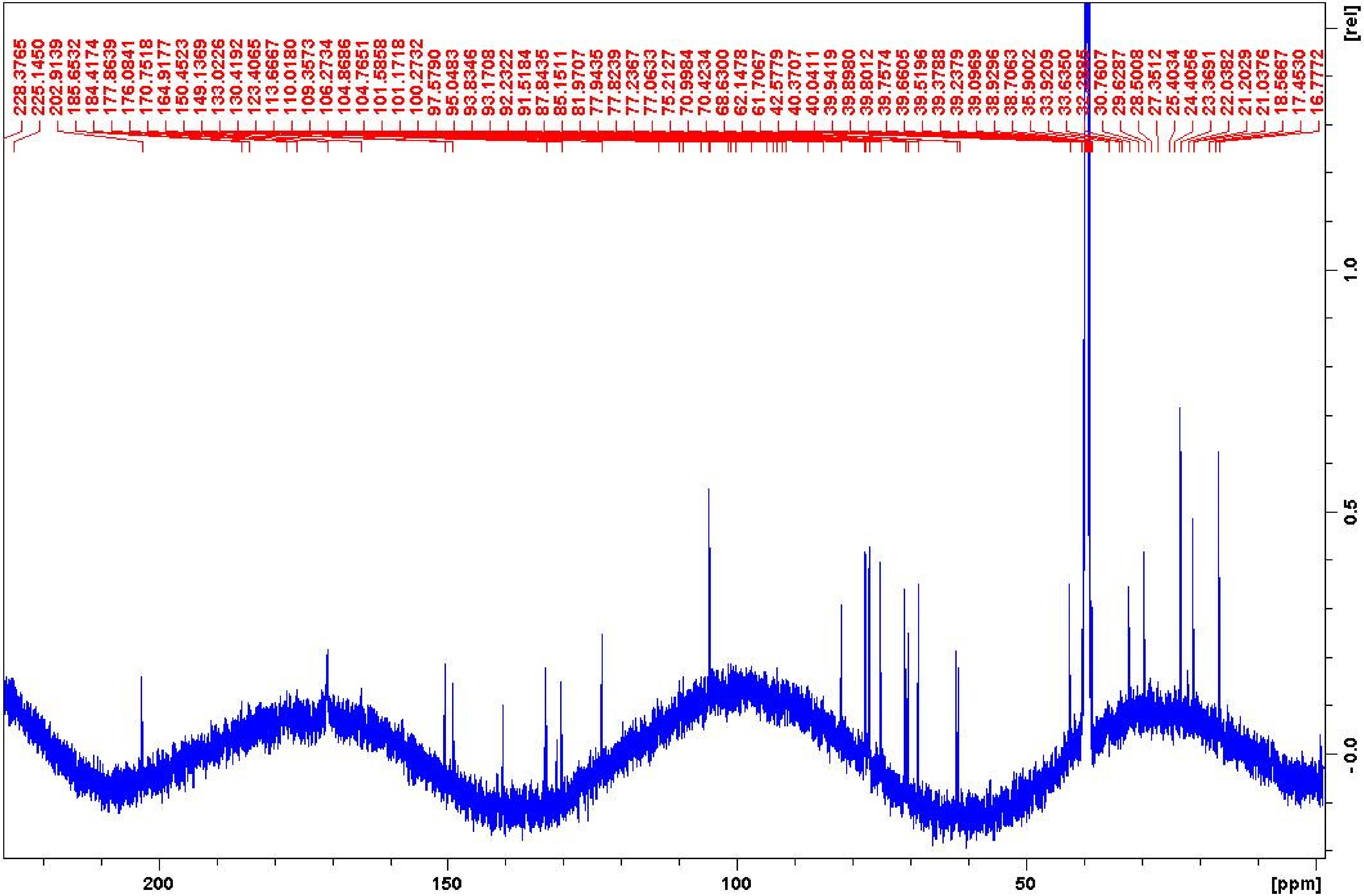

Figure S8-C. $^{13}C$ NMR spectrum of compound **2** in $D_2O$.

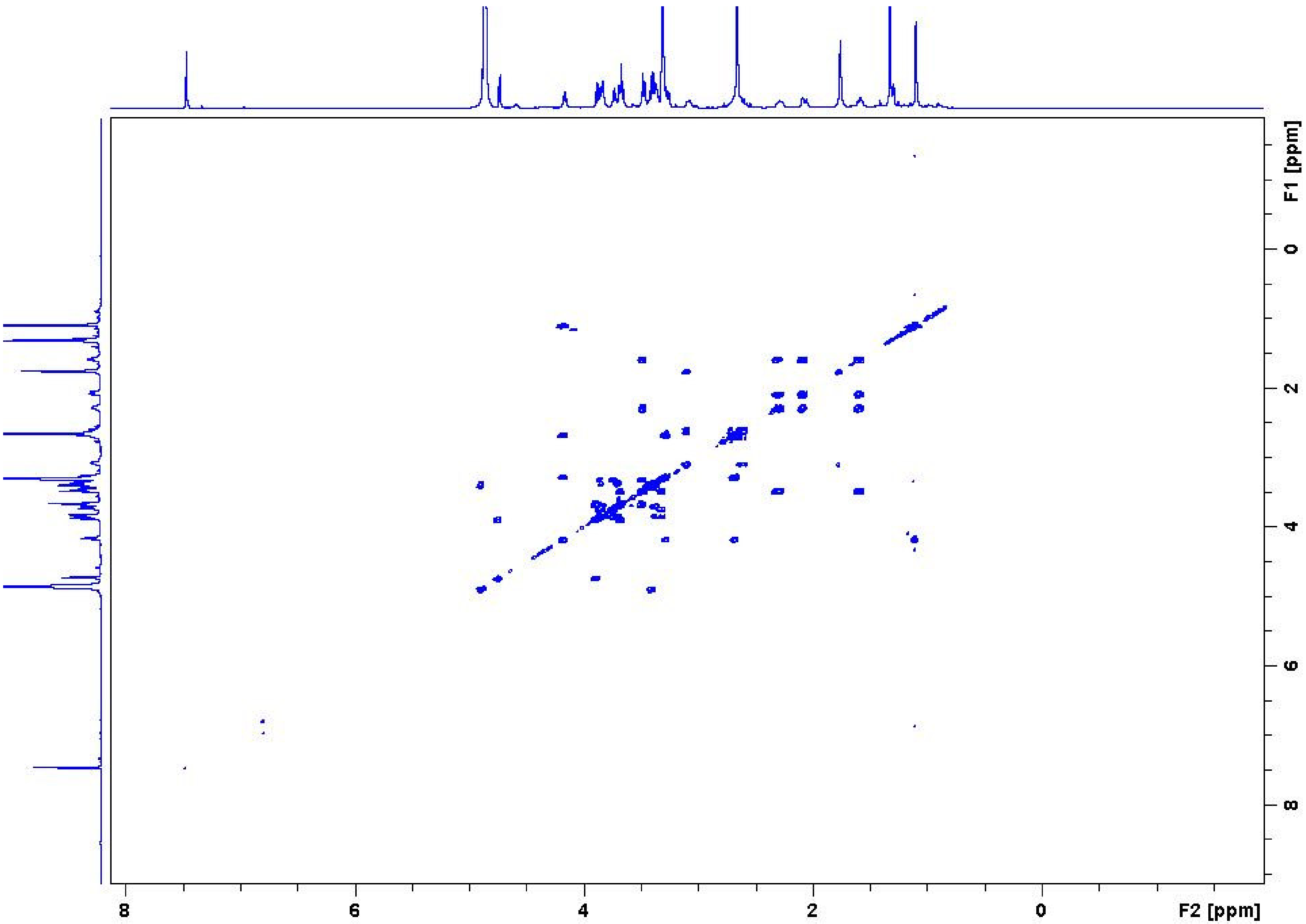


Figure S9-A. COSY spectrum of compound **2** in Methanol-$d_4$.

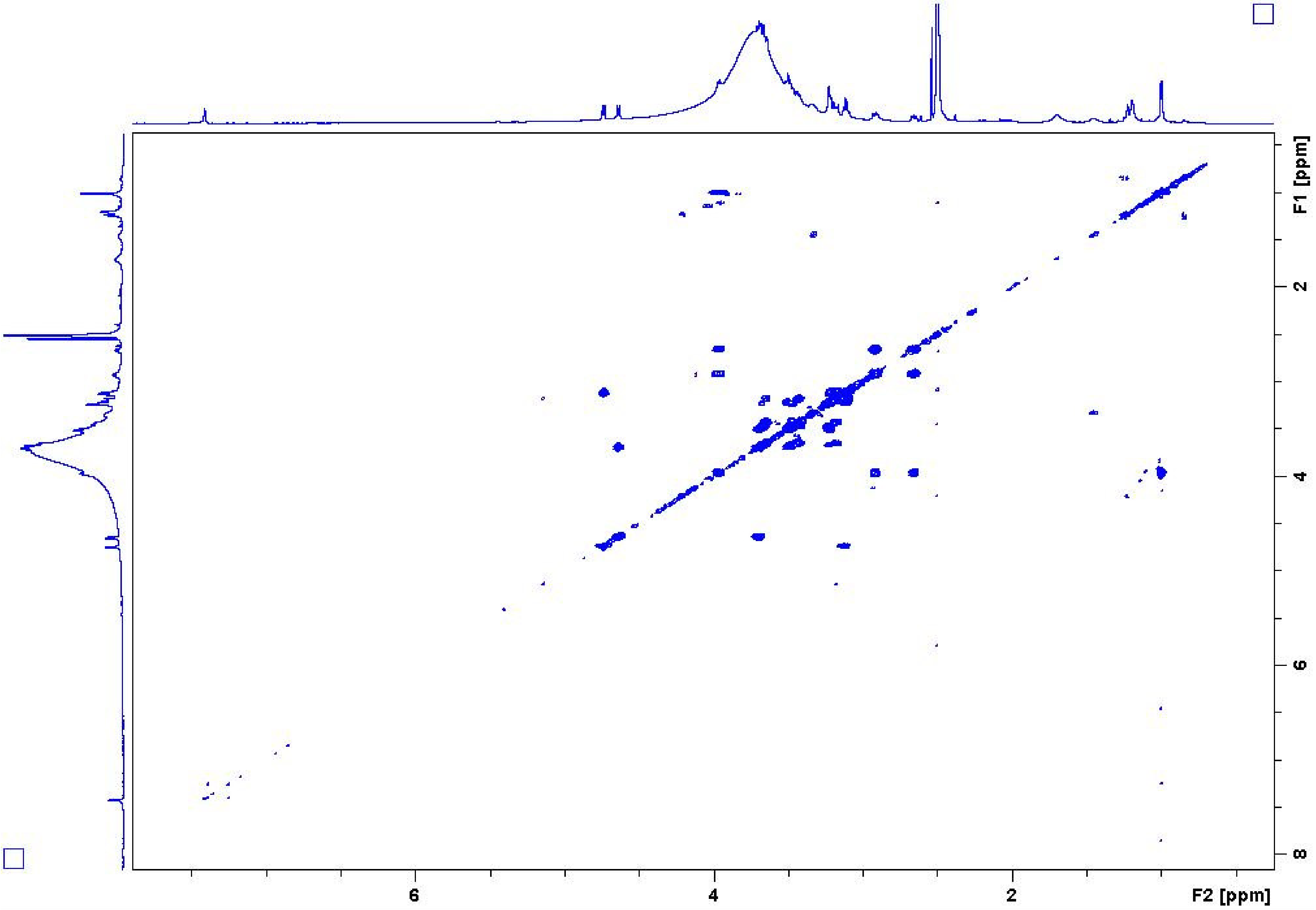


Figure S9-B. COSY spectrum of compound **2** in DMSO-$d_6$.

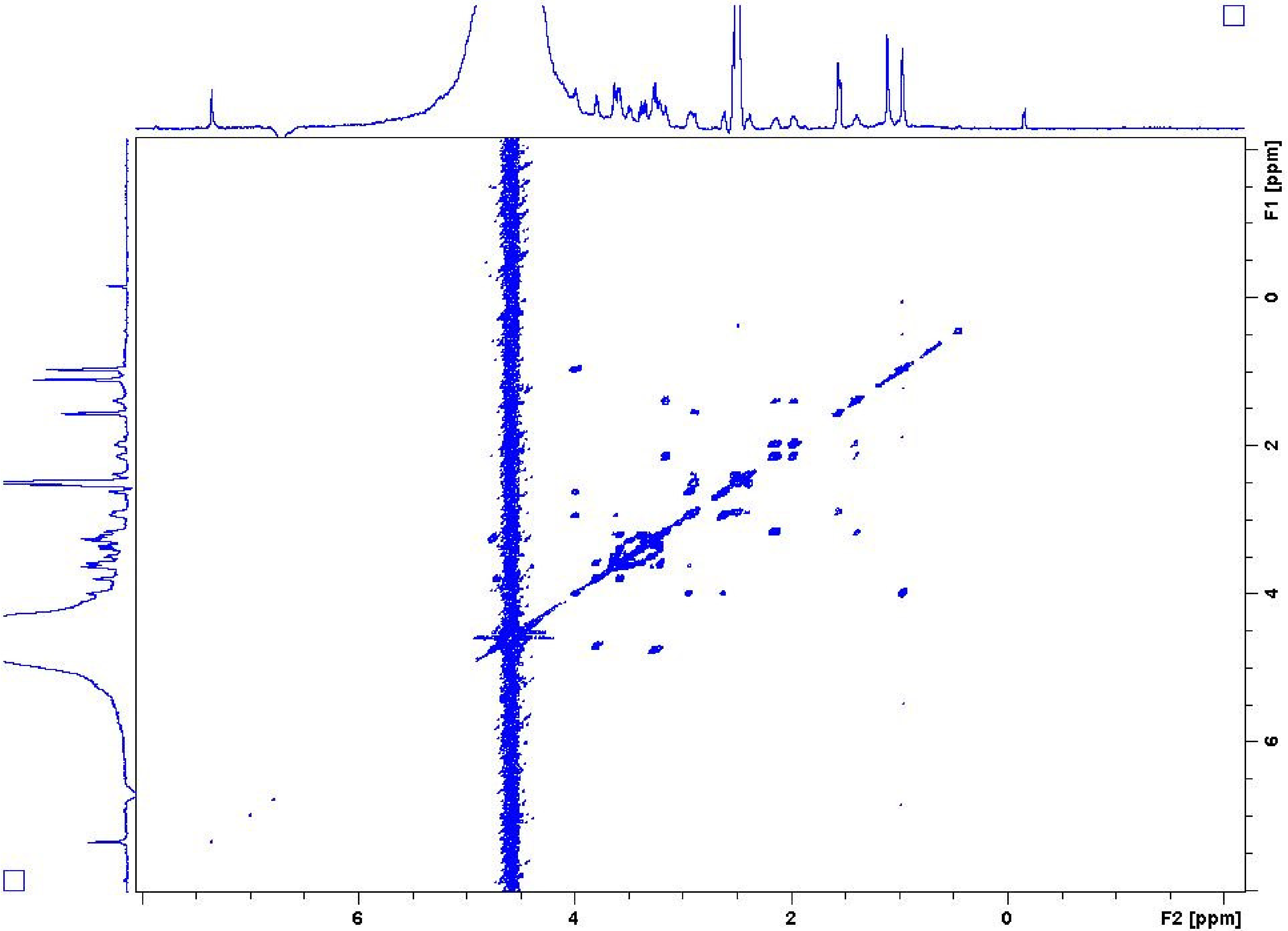


Figure S9-C. COSY spectrum of compound **2** in $D_2O$.

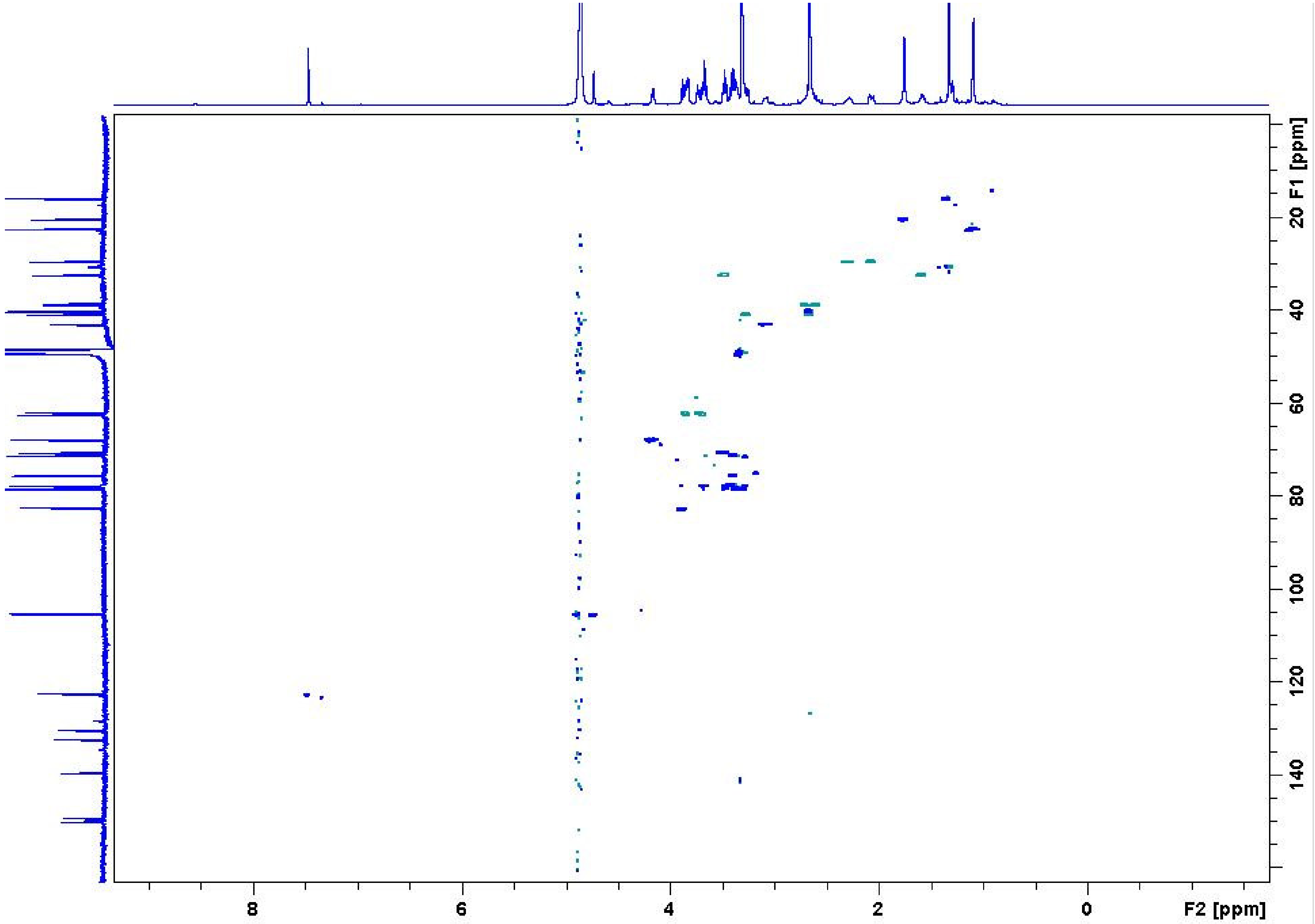


Figure S10-A. HSQC spectrum of compound **2** in Methanol-$d_4$.

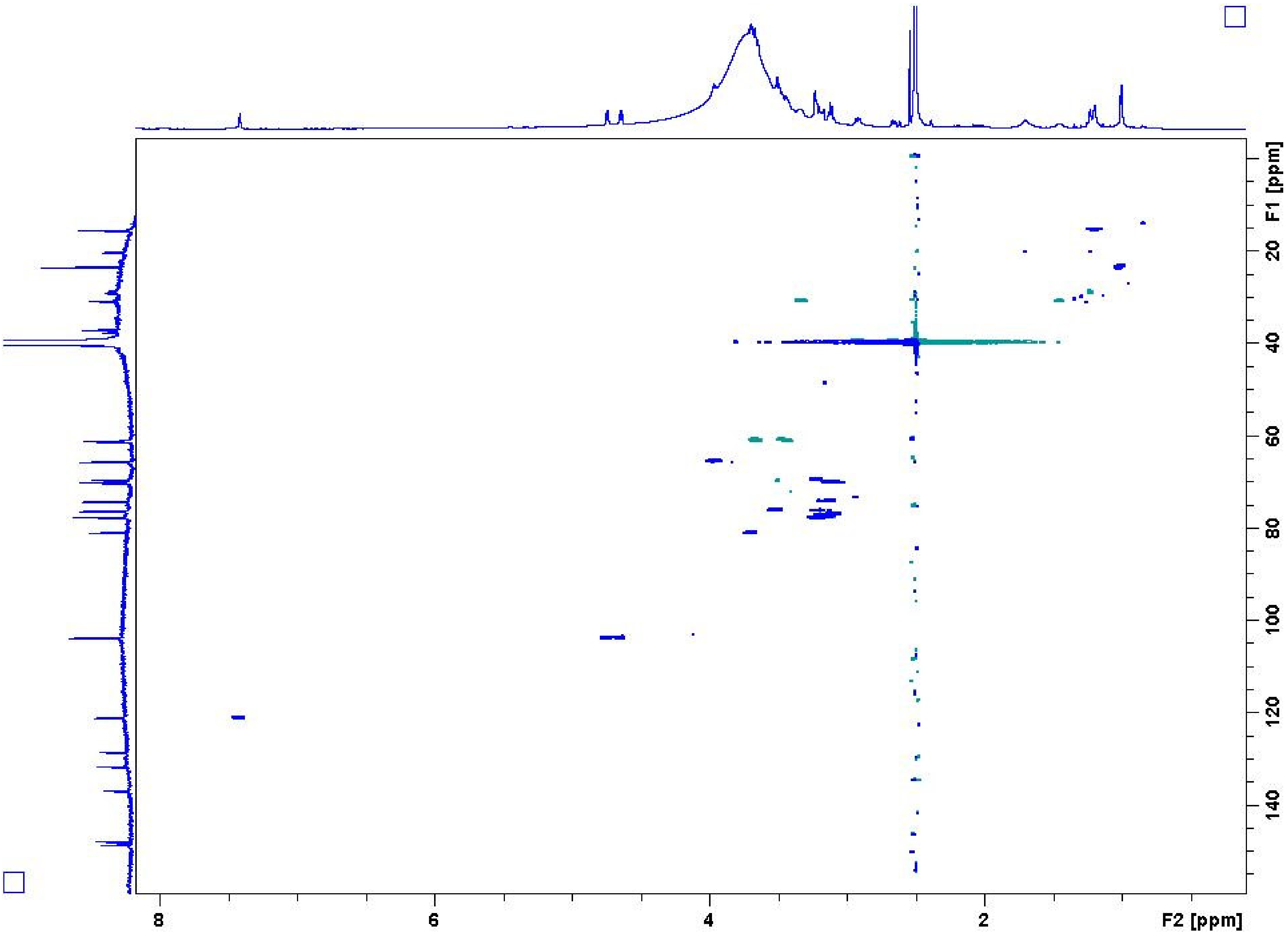


Figure S10-B. HSQC spectrum of compound **2** in DMSO-$d_6$.

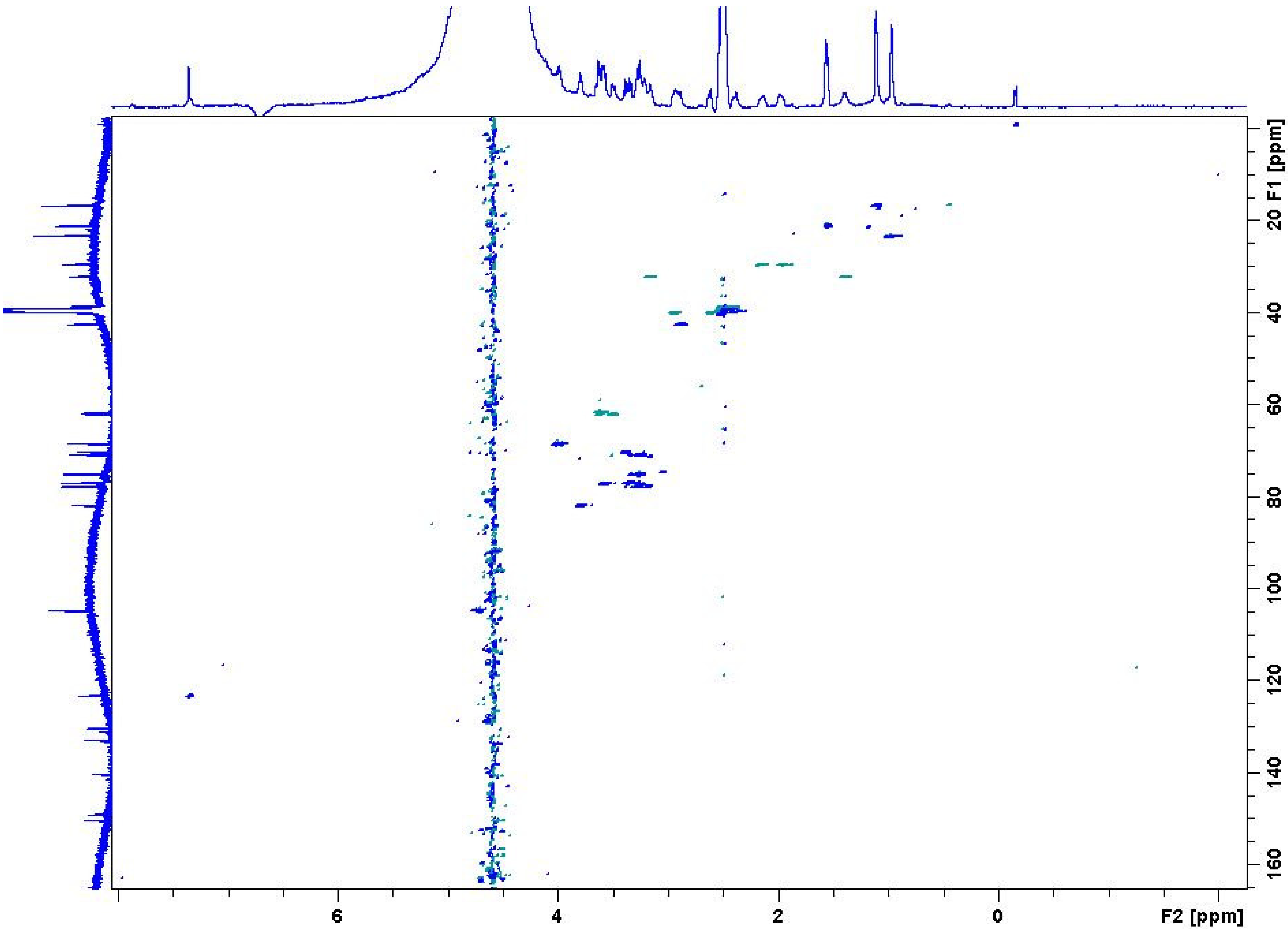

Figure S10-C. HSQC spectrum of compound **2** in $D_2O$.

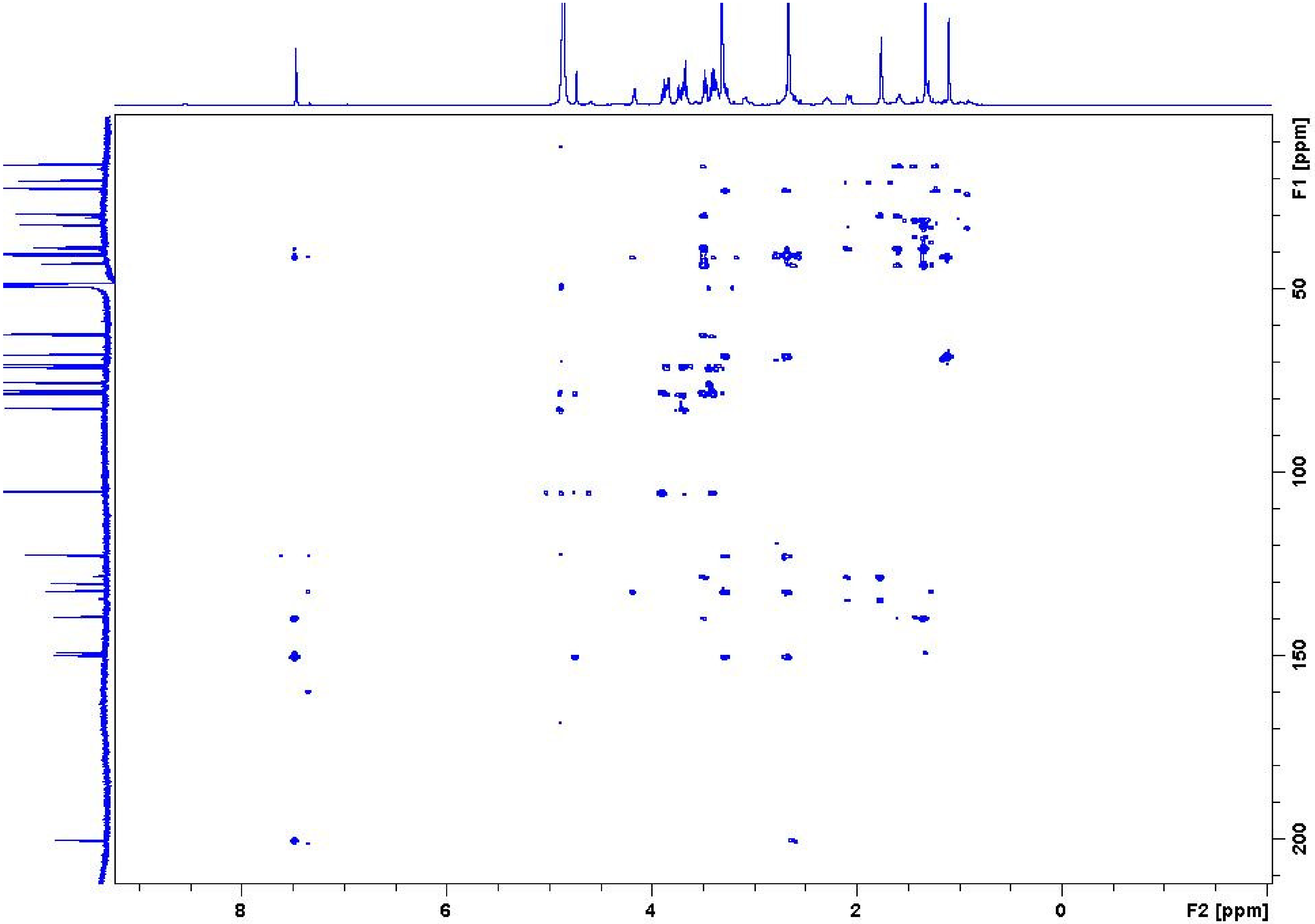


Figure S11-A. HMBC spectrum of compound **2** in Methanol-$d_4$.

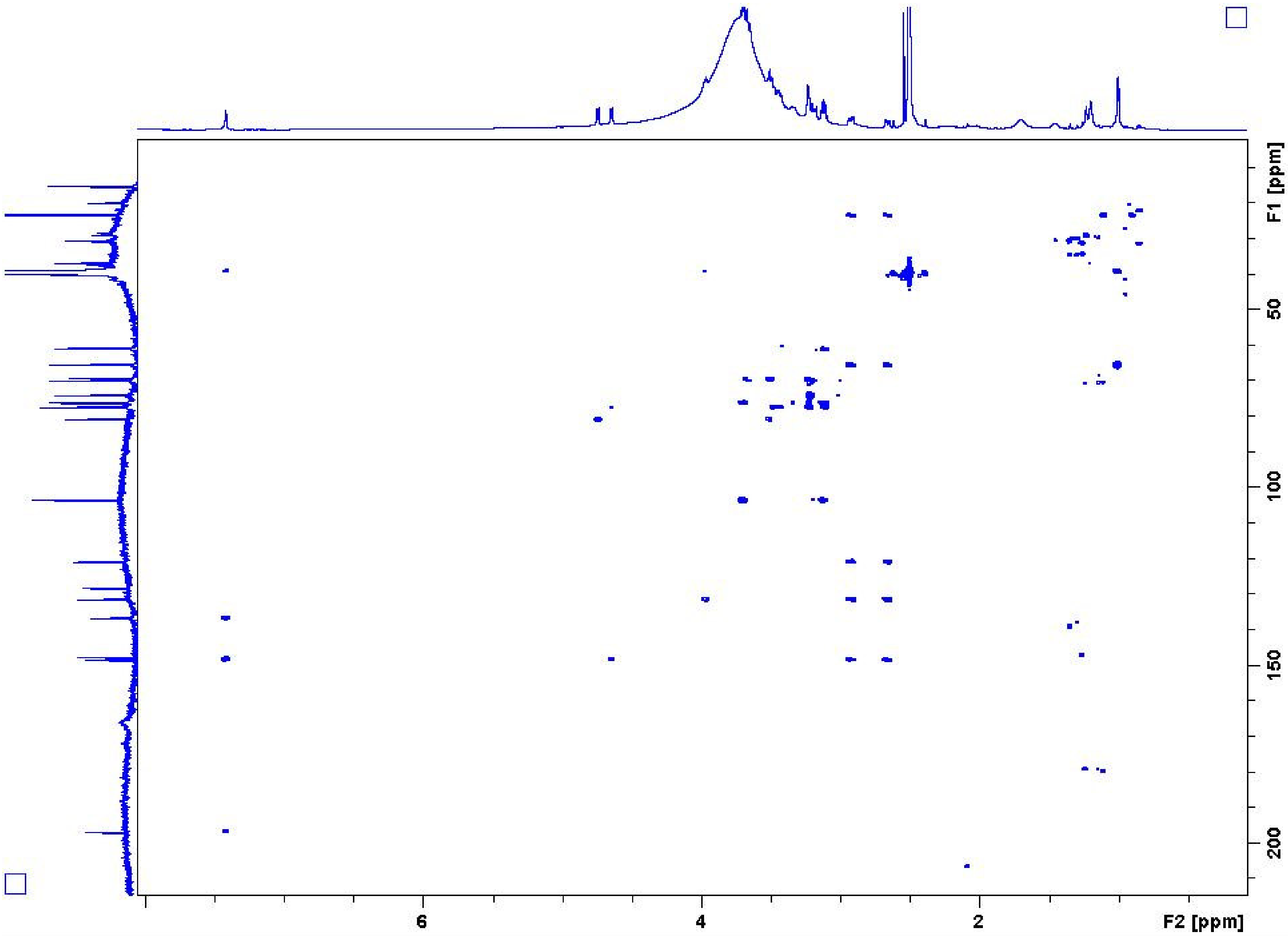


Figure S11-B. HMBC spectrum of compound **2** in DMSO-$d_6$.

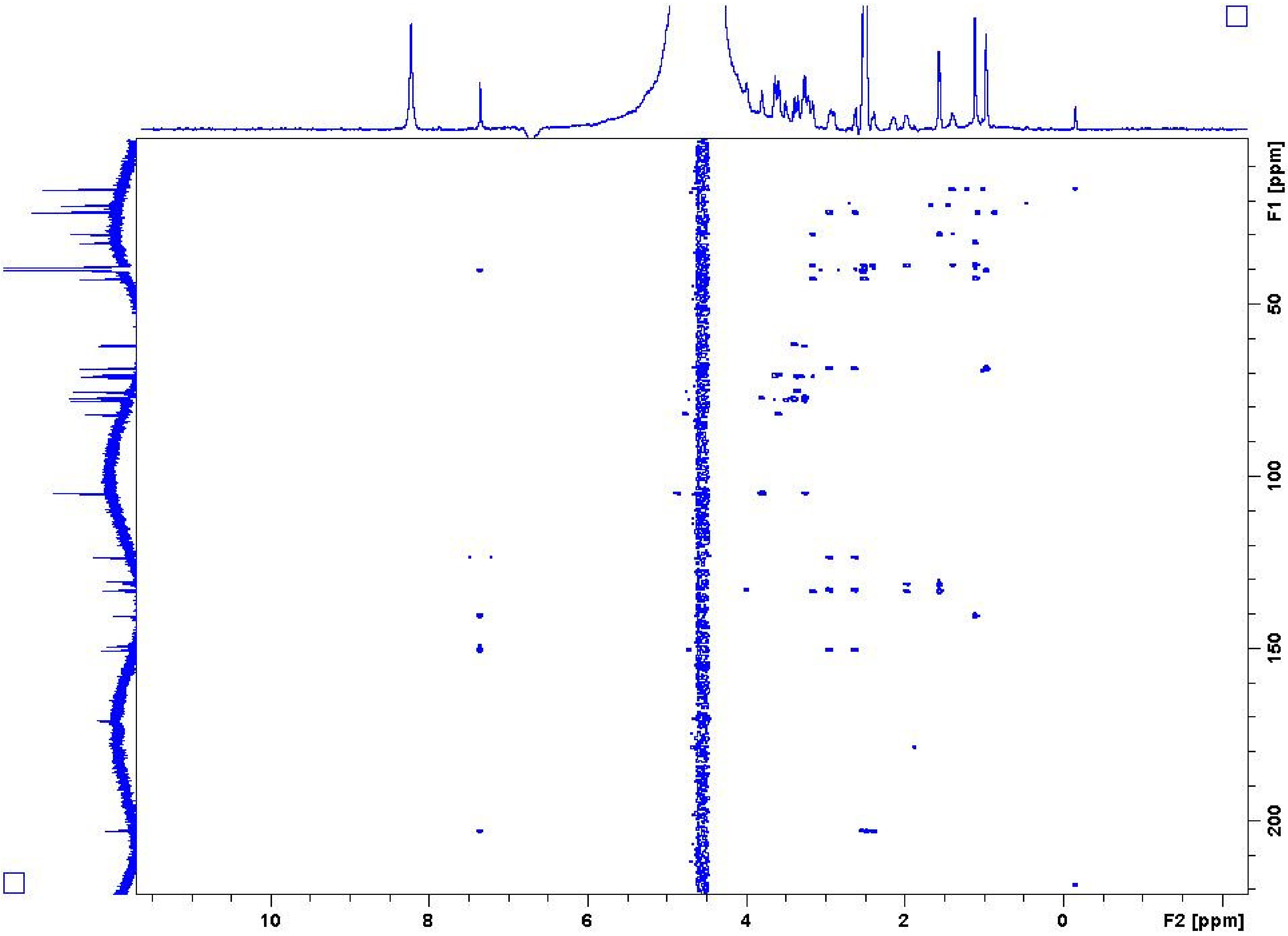


Figure S11-C. HMBC spectrum of compound **2** in $D_2O$.

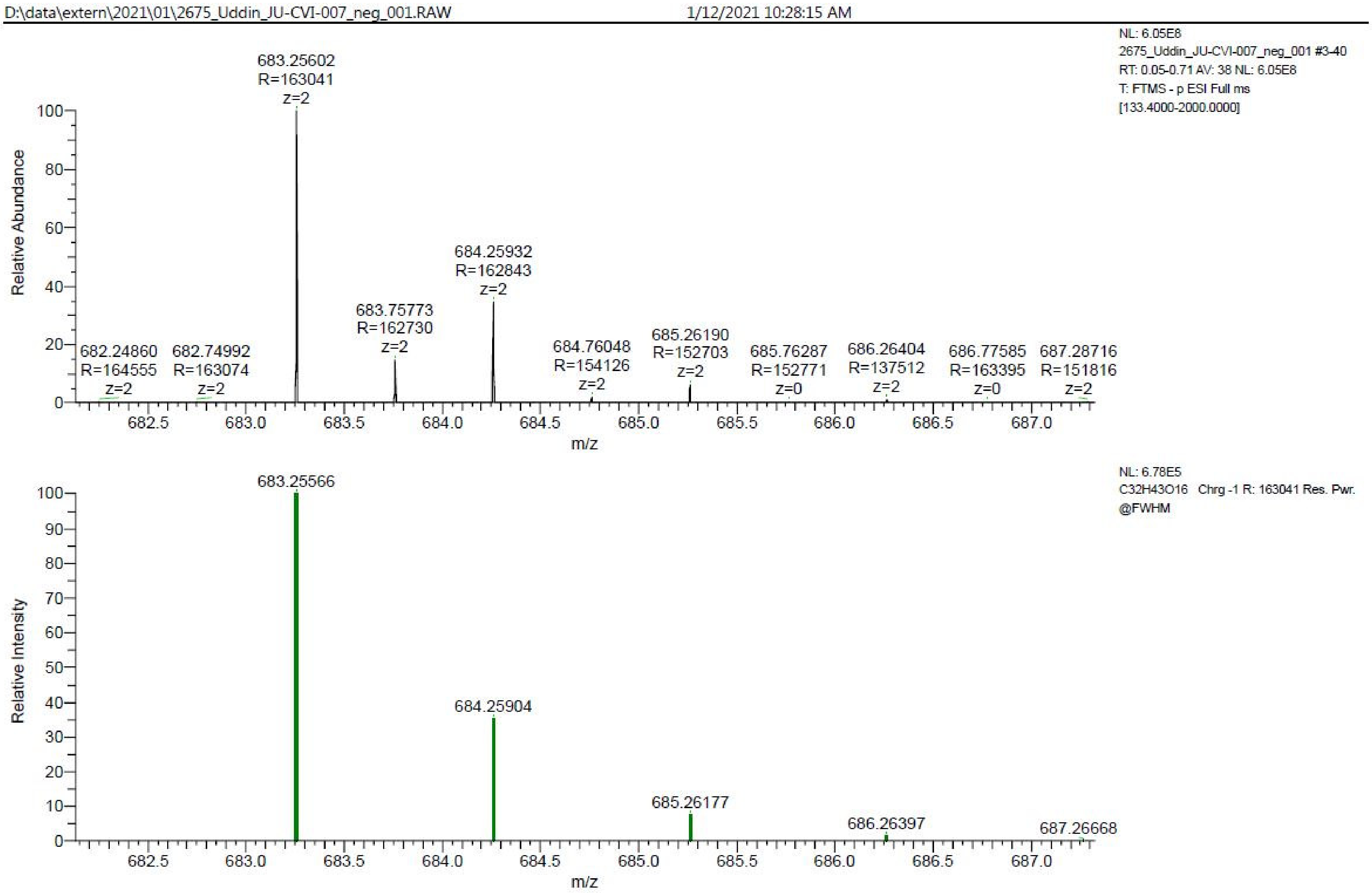


| Peak Mass | Display Form... | Combined Fit | RDB | Delta [ppm] | Theo. mass | Rank | Combined Sc... | # Matched Iso. | # Missed Iso. | MS Cov. [%] | Pattern Cov. [... | MSMS Match... |
|---|---|---|---|---|---|---|---|---|---|---|---|---|
| 683.25602 | $C_{32}H_{43}O_{16}$ | 100 | 11.50 | 0.54 | 683.25566 | 1 | 0 | 6 | 0 | 0 | 0 | (Collection) |

Figure S12. HR mass spectrum of compound **2** in methanol.

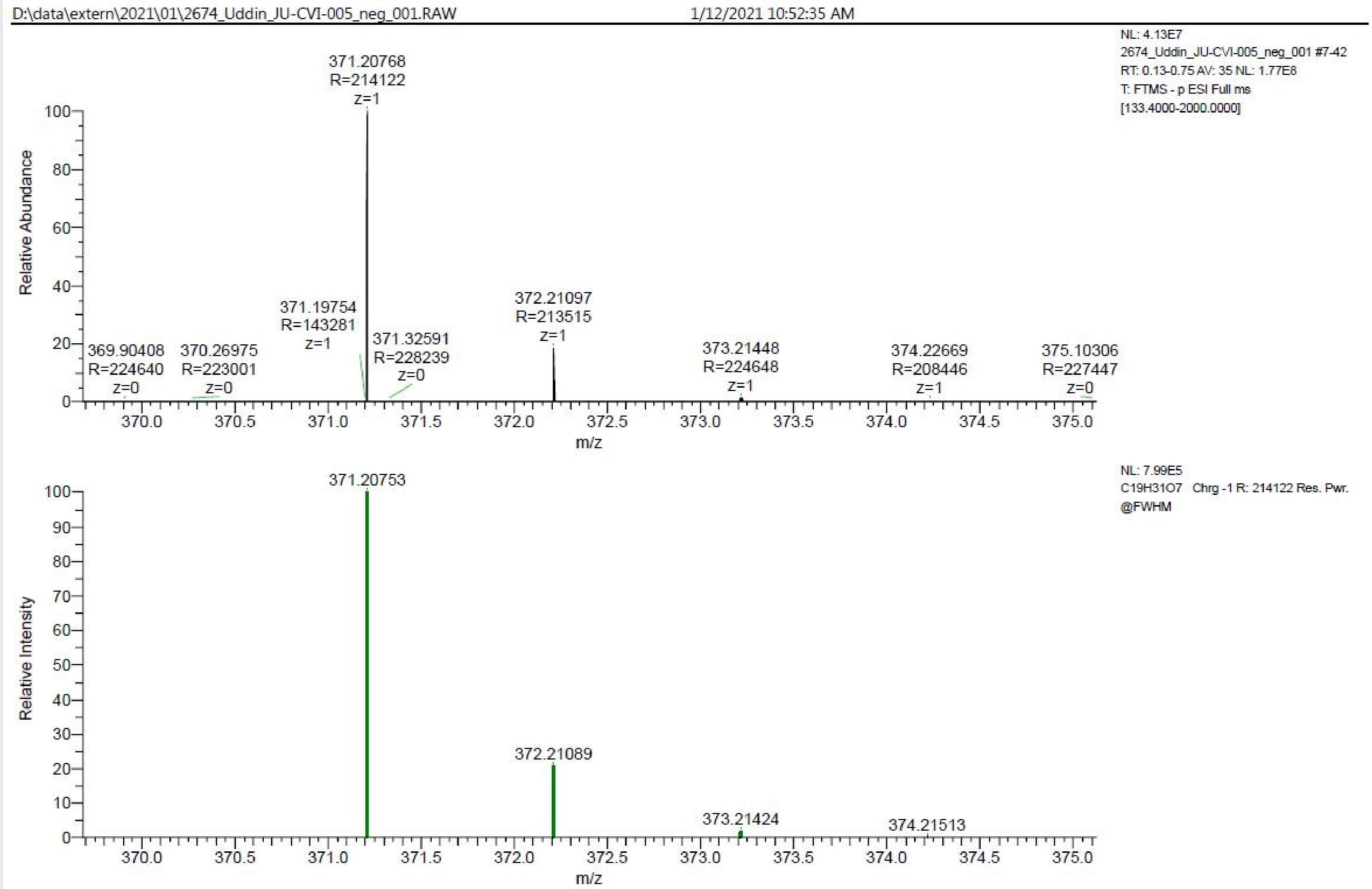


| Peak Mass | Display Form... | Combined Fit | RDB | Delta [ppm] | Theo. mass | Rank | Combined Sc... | # Matched Iso. | # Missed Iso. | MS Cov. [%] | Pattern Cov. [... | MSMS Match... |
|---|---|---|---|---|---|---|---|---|---|---|---|---|
| 371.20768 | $C_{19}H_{31}O_7$ | 100 | 4.50 | 0.42 | 371.20753 | 1 | 0 | 5 | 2 | 0 | 0 | (Collection) |

Figure S13. HR mass spectrum of compound **3** in methanol.

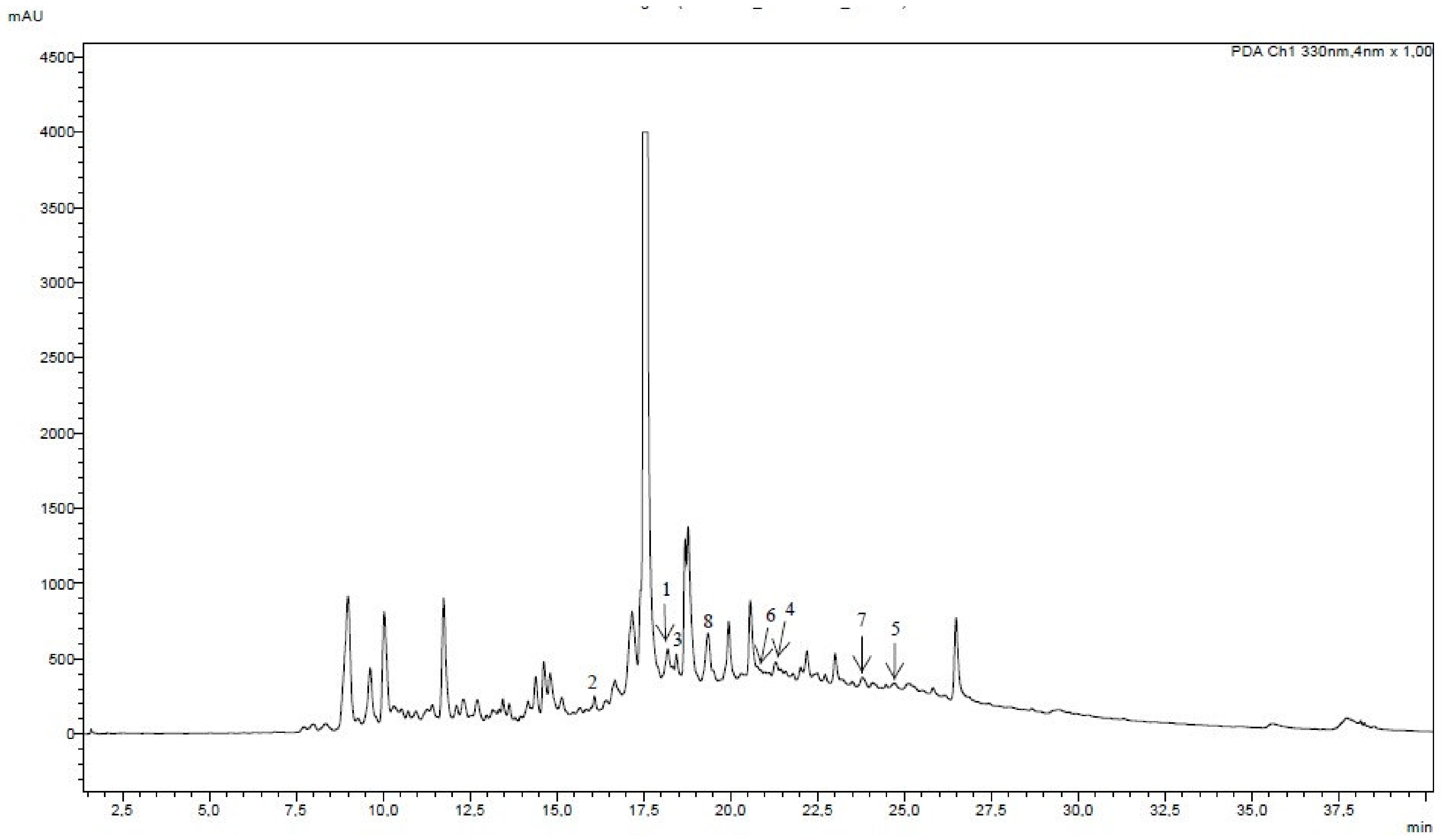


Figure S14. UHPLC (λ = 330 nm) chromatogram of *n*-butanol fraction from leaves of *Clerodendrum infortunatum.*

Table S1. 1D ($^1$H and $^{13}$C) NMR spectroscopic data for compound **3**

| | **3**[a] | **3**[b] | **3**[a] | **3**[b] |
|---|---|---|---|---|
| Position | $\delta_C$, type | $\delta_C$, type | $\delta_H$ ($J$ in Hz) | $\delta_H$ ($J$ in Hz) |
| cyclohexanone moiety | | | | |
| 1 | 37.3, C | 35.9, C | | |
| 2 | 48.1, $CH_2$ | 47.0, $CH_2$ | 1.97, d (17.5)<br>2.46, d (17.5) | 1.87, d (17.5)<br>2.34, d (17.5) |
| 3 | 202.4, C | 198.1, C | | |
| 4 | 125.4, CH | 124.2, CH | 5.80, s | 5.72, s |
| 5 | 170.1, C | 166.1, C | | |
| 6 | 52.4, CH | 50.1, CH | 1.99, m[b] | 1.90, t (5.0) |
| 7 | 26.8, $CH_2$ | 25.1, $CH_2$ | 1.50, m<br>1.98, m[b] | 1.38, m<br>1.78, m |
| 8 | 37.8, $CH_2$ | 36.2, $CH_2$ | 1.61, m<br>1.67, m | 1.50, m |
| 9 | 75.5, CH | 73.3, CH | 3.88, m | 3.74, dd (12.0, 6.0) |
| 10 | 19.9, $CH_3$ | 19.5, $CH_3$ | 1.18, d (6.0) | 1.1, d (6.0) |
| 11 | 29.1, $CH_3$ | 26.8, $CH_3$ | 1.01, s | 1.00, s |
| 12 | 27.5, $CH_3$ | 28.5, $CH_3$ | 1.09, s | 0.97, s |
| 13 | 25.0, CH | 24.1, CH | 2.05, d (1.0) | 1.97, m |
| glucopyranosyl moiety | | | | |
| 1′ | 102.1, CH | 100.7, CH | 4.32, d (8.0) | 4.16, d (8.0) |
| 2′ | 75.2, CH | 73.5, CH | 3.14, dd (9.0, 8.0) | 2.89, t (8.5) |
| 3′ | 78.2, CH | 76.9, CH | 3.35, dd (9.0, 9.0) | 3.12, t (8.5) |
| 4′ | 71.8, CH | 70.3, CH | 3.26, m | 3.03, m |
| 5′ | 77.9, CH | 76.8, CH | 3.25, m | 3.05, m |
| 6′ | 62.9, $CH_2$ | 61.3, $CH_2$ | 3.64, dd (12.00, 5.5)<br>3.85, dd (12.00, 2.0) | 3.41, m<br>3.64, m |

[a]Spectra were referenced to solvent residual and solvent signals of $CD_3OD$ at 3.31 ppm ($^1$H NMR, 600 MHz) and 49.0 ppm ($^{13}$C NMR, 150 MHz), respectively.

[b]Spectra were referenced to solvent residual and solvent signals of $(CD_3)_2SO$ at 2.50 ppm ($^1$H NMR, 600 MHz) and 39.52 ppm ($^{13}$C NMR, 150 MHz), respectively.
